# Bioinspired Zwitterionic Block Polymer-Armored Nitric Oxide-Generating Coating Combats Thrombosis and Biofouling

**DOI:** 10.34133/research.0423

**Published:** 2024-08-01

**Authors:** Qing Ma, Wentai Zhang, Xiaohui Mou, Nan Huang, Haimang Wang, Hongyu Zhang, Zhilu Yang

**Affiliations:** ^1^ School of Materials Science and Engineering, Key Lab of Advanced Technology of Materials of Education Ministry, Southwest Jiaotong University, Chengdu, 610031, China.; ^2^ Dongguan Key Laboratory of Smart Biomaterials and Regenerative Medicine, The Tenth Affiliated Hospital, Southern Medical University, Dongguan, 523059, China.; ^3^ GuangZhou Nanchuang Mount Everest Company for Medical Science and Technology, Guangzhou, 510670, China.; ^4^ State Key Laboratory of Tribology in Advanced Equipment, Department of Mechanical Engineering, Tsinghua University, Beijing, 100084, China.; ^5^ Wenzhou Institute, University of Chinese Academy of Sciences, Wenzhou, 352001 Zhejiang, China.

## Abstract

Thrombosis and infection are 2 major complications associated with central venous catheters (CVCs), resulting in substantial mortality and morbidity. The concurrent long-term administration of antibiotics and anticoagulants to address these complications have been demonstrated to cause severe side effects such as antibiotic resistance and bleeding. To mitigate these complications with minimal or no drug utilization, we developed a bioinspired zwitterionic block polymer-armored nitric oxide (NO)-generating functional coating for surface modification of CVCs. This armor was fabricated by precoating with a Cu-dopamine (DA)/selenocysteamine (SeCA) (Cu-DA/SeCA) network film capable of catalytically generating NO on the CVCs surface, followed by grafting of a zwitterionic p(DMA-*b*-MPC-*b*-DMA) polymer brush. The synergistic effects of active attack by NO and copper ions provided by Cu-DA/SeCA network and passive defense by zwitterionic polymer brush imparted the CVCs surface with durable antimicrobial properties and marked inhibition of platelets and fibrinogen. The in vivo studies confirmed that the surface-armored CVCs could effectively reduce inflammation and inhibit thrombosis, indicating a promising potential for clinical applications.

## Introduction

In clinical, central venous catheters (CVC_S_) are pivotal for the prolonged administration of nutrition support, drug delivery, diagnostics, and chemotherapy [1]. However, the primary challenge of CVCs lies in the severe complications such as infections and thrombosis [2]. This issue arises from the introduction of foreign materials, which invariably become sites for microbial accumulation and blood coagulation [3]. Bacteria can colonize on the surface of the material, forming a biofilm that leads to intractable infections [4]. These infections can activate coagulation pathways, further promoting thrombus formation. Bacterial endotoxins and proinflammatory cytokines can directly or indirectly activate platelets, resulting in coagulation system dysregulation [5]. On the other hand, the production of fibrous sheaths in blood-contacting devices also provides suitable conditions for bacterial colonization, increasing the risk of infection [6]. This scenario has necessitated the sustained systemic use of antibiotics and anticoagulants over the past decades to mitigate these issues. However, the prolonged and excessive use of these medications can lead to adverse reactions, such as fever, antibiotic resistance, uncontrollable hemorrhage, and thrombocytopenia [7]. This complexity of adverse effects and the detrimental impact of anticoagulants and antibiotics signify the need to develop an alternative method to reduce catheter dysfunction and minimize associated harm.

In recent years, surface engineering technologies have gained heightened attention in the field of blood-contacting medical devices [8,9]. These techniques aim to enhance and modify the surface of the devices to impart distinct functionalities, such as anticoagulant and antimicrobial properties. The adoption of these advancements has shown considerable benefits in addressing clinical challenges related to implanted devices [10,11]. Currently, there are 2 mainstream surface modification methods for antibacterial and anticoagulant properties including (a) passive defense [12], employing ultrahydrophobic polymers like perfluorinated polymers [13], hydrophilic polymers such as hyaluronic acid [14], and zwitterionic polymers [15] to reduce protein adherence and bacterial colonization and (b) active attack [12], wherein bioactive molecules with antimicrobial or anticoagulant properties such as cationic polymers [16], metal ions [17], heparin [18], or peptides [19,20] are loaded or grafted onto the surface. Nonetheless, these strategies continue to face challenges in controlling the adsorption of blood proteins and bacteria on the surfaces of medical devices. For instance, passive antifouling surfaces may lose effectiveness over time in complex pathological environments [21]. Similarly, regarding active attack strategies, the surface concentration of bioactive molecules might deplete or deteriorate, diminishing their long-term antimicrobial and anticoagulant efficacy [22]. Therefore, the existing strategies, depending solely on either one-sided passive defense or active attack, face challenges in maintaining the surfaces with long-lasting, effective anticoagulant and antibacterial properties.

**Fig. 1.**
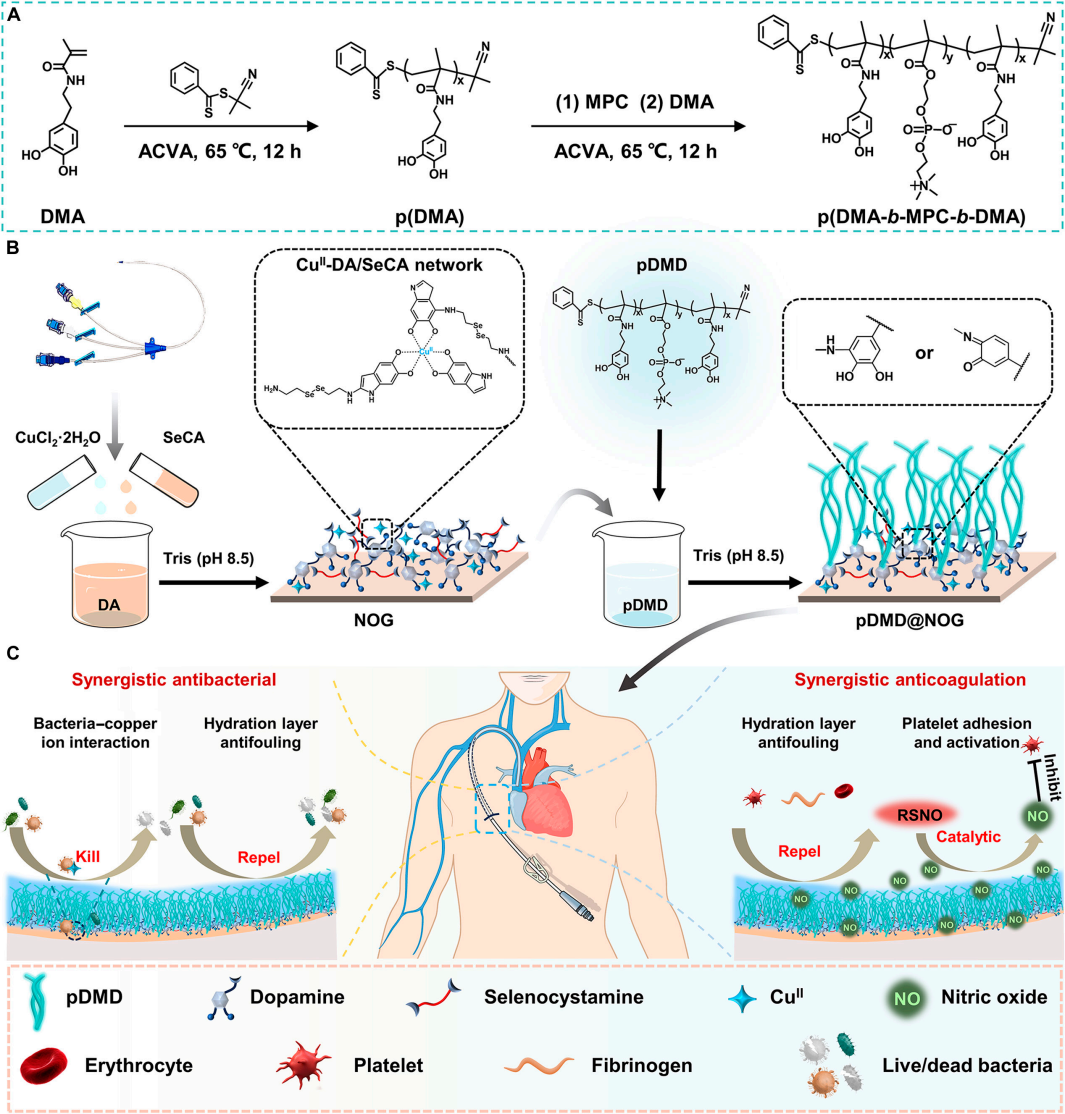
Schematic diagram showing the design principle of NO-generating zwitterionic block polymer armor and its application in indwelling CVCs. (A) Schematic illustration for the synthesis of pDMD. (B) Preparation of NO release surface functionalization through metal–phenol–amine chemistry followed by zwitterionic polymer grafting via catecholamine chemistry. (C) Schematic diagram of the synergistic anticoagulant and antibacterial abilities of the pDMD@NOG armor.

In this context, we proposed a bioinspired zwitterionic block polymer-armored nitric oxide (NO) generating coating that combines passive defense with aggressive attack strategies, endowing the CVCs with durable antifouling, antimicrobial, and anticoagulant properties (Fig. 1). Through a simple one-step molecular/ion coassembly process, we developed a metal-phenol-(amine) network (MPAN) coating with exceptional adhesion and universal substrate applicability on the catheter surface [23]. This coating comprised selenocystamine (SeCA), which catalyzed NO release, copper ions for its potent bactericidal activity and further catalyzing NO release, and dopamine (DA) to improve adhesion, serving as the aggressive attack element. NO prevented platelet adhesion by increasing cyclic guanosine monophosphate (cGMP), and copper ions disrupted bacterial cell membranes to provide potent bactericidal action [24]. To mitigate the diminution of coating functionality due to the inevitable adsorption of bacteria and proteins on the active attack surface, we incorporated an additional protective layer. We synthesized a block dual-biomimetic copolymer p(DMA-*b*-MPC-*b*-DMA) (pDMD) using adhesive dopamine methacrylamide (DMA) and 2-methacryloyloxyethyl phosphorylcholine (MPC, a biomimetic zwitterionic molecule that emulated a major component of cell membranes) [25]. Utilizing the abundant primary amine groups provided by MPAN, stable superhydrophilic zwitterionic polymer brushes were formed through phenolic amine chemical crosslinking. This strategic combination of proactive attack (releasing NO and copper ions) and passive defense (superhydrophilic pDMD polymer brush) was anticipated to effectively enhance the antimicrobial and anticoagulant functions of the surface, potentially surpassing the current single-function surfaces.

## Results

### Fabrication and characterization of pDMD block polymer and pDMD@NOG armor

Utilizing reversible addition-fragmentation chain transfer (RAFT) polymerization, we prepared a novel block polymer comprising zwitterionic choline phosphate, with antifouling properties, and DA, endowing the polymer with adhesive performance and secondary reactivity. ^1^H nuclear magnetic resonance (NMR) spectrum of pDMD revealed that the peaks at 2.84, 3.01, 6.82, and 7.92 ppm corresponded to groups in DMA, while the peaks at 3.26, 3.71, 4.11, and 4.32 ppm were assigned to groups in MPC (Fig. S1A). Gel permeation chromatography (GPC) analysis result, presented in Fig. S1B, indicated that the pDMD block polymer had a molecular weight of 258.7 kDa and a polydispersity index of 1.09. Both ^1^H NMR and GPC analysis confirmed the successful synthesis with uniform molecular weight. Additionally, ultraviolet (UV) spectroscopy result demonstrated the retention of catechol groups in the polymer pDMD, as evidenced by absorption at 281 nm, similar to that of DA (Fig. S2). The absence of peaks above 300 nm indicated that the catechol groups remained unoxidized [26,27].

**Fig. 2.**
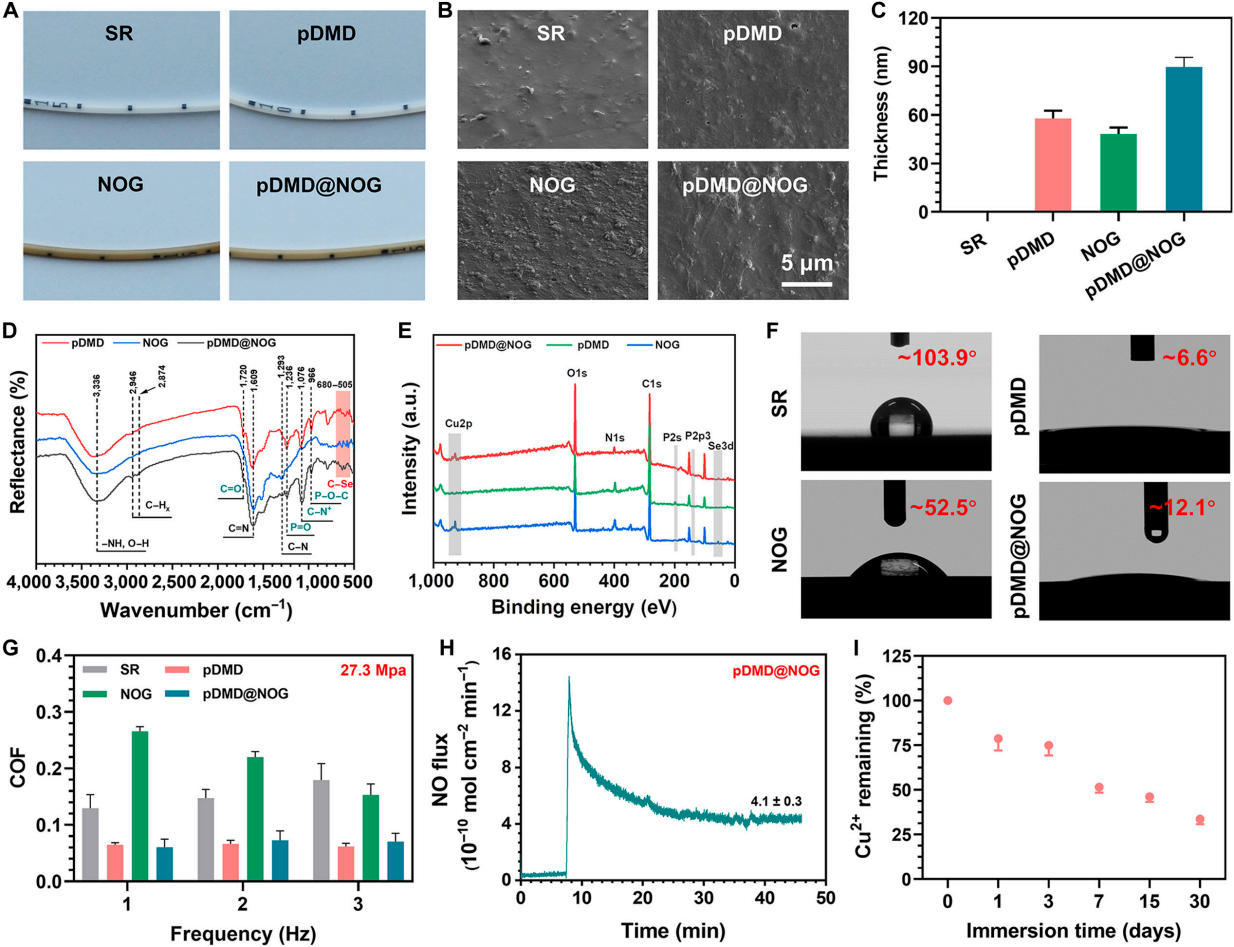
Characterization of functional pDMD@NOG armor on SR substrate. (A) Representative photographs and (B) SEM images of SR, pDMD, NOG, and pDMD@NOG. (C) Evolution of pDMD@NOG thickness on SR substrates determined by ellipsometry measurements. (D) FTIR and (E) XPS spectra of SR substrates before and after modification by pDMD, NOG, and pDMD@NOG. (F) WCA measurement of bare and modified SR. (G) COF-frequency plots illustrating the interaction of PS microspheres with various surfaces under a under a consistent pressure (27.3 MPa) and 3 different frequencies (1, 2, and 3 Hz). (H) Real-time release of NO by pDMD@NOG measured in PBS supplemented with 10 μM GSH and 10 μM GSNO at 37°. (I) Copper ion concentrations were assessed at various times using inductively coupled plasma mass spectrometry.

Drawing inspiration from the catecholamine structure unit found in mussel adhesive proteins, DA-assisted deposition technology in surface chemistry has been increasingly recognized for its outstanding adhesion properties, substrate versatility, and biocompatibility [28,29]. In this study, a NO-releasing coating with active anticoagulant and antibacterial properties was successfully constructed on a silicone rubber (SR) substrate using Cu^2+^, DA, and SeCA, and the polymer pDMD was then grafted via catecholamine chemistry. This process enabled the SR surface to exhibit both active anticoagulant and antibacterial properties, as well as passive antifouling capabilities. As a control, passive defense (i.e., pDMD) and active attack (i.e., NOG) coatings were prepared separately. After deposition, the CVCs color transitioned from milky white to polyphenolic dark brown (Fig. 2A), signifying the formation of the coating armor on the surface. This observation was further confirmed by scanning electron microscopy (SEM) analysis, showing that both the inner and outer surfaces of the CVC were coated with uniform and continuous armor (Fig. 2B and Fig. S3) with a thickness of 95 nm (Fig. 2C). The chemical structure and composition changes of SR surface in each modified step were further evaluated through Fourier transform infrared (FTIR) spectroscopy and x-ray photoelectron spectroscopy (XPS). Figure 2D shows that characteristic peaks of the polymer MPC (C=O, P=O, C–N^+^, and P–O–C at 1,720, 1,236, 1,076, and 966 cm^−1^, respectively) were detected in the pDMD@NOG armor. Additionally, stretching vibration peaks of the C–Se bond appeared in the range of 680 to 505 cm^−1^, indicating the successful preparation of the armor on the substrate surface. Furthermore, aromatic C=N and aromatic C–N peaks were observed at 1,458 and 1,280 cm^−1^, respectively, indicating the involvement of phenol-amine crosslinking reactions (i.e., Michael addition and Schiff base reaction) in the formation of the pDMD@NOG armor. XPS analysis further substantiated the successful creation of the coating, as evidenced by the detection of characteristic elements, including phosphorus (P), copper (Cu), and selenium (Se) (Fig. 2E).

Surface grafting with zwitterion polymer markedly enhanced the hydrophilic properties of the material and altered the adhesion capabilities of substances on its surface [30]. Crucially, the hydration layer formed by the interaction of MPC molecules and water molecules could effectively reduce the friction coefficient of the material surface, thereby imparting improved lubricity to the material. This reduction in friction could minimize potential implantation-related injuries between CVCs and blood vessels [31]. The water contact angles (WCAs) of pDMD and pDMD@NOG were significantly lower at 6.6° and 12.1°, respectively, compared to the SR surface with a WCA of 103.9° (Fig. 2F), effectively enhancing the hydrophilic property of the material surface. The slight variation in the WCA may be attributed to the minimal release of copper ions binding with phosphate ions during the grafting process [32]. Subsequently, the values of coefficient of friction (COF) of the material surface were determined under different loads, confirming these results. For instance, at a pressure of 23.7 MPa, the COF of pDMD@NOG decreased by approximately 52.7% compared to that of SR. Notably, the COF of the pDMD@NOG armor remained stable under different loads and scan frequencies (Fig. 2G and Fig. S4A and B), indicating that the pDMD@NOG armor was easier to slide during catheter implantation. Chemiluminescence measurements were performed to evaluate the NO catalytic behavior of both NOG and pDMD@NOG-coated SR substrates. This follows our prior research, which established that the release of NO could significantly reduce platelet adhesion and activation, thereby providing anticoagulant benefits. Furthermore, optimal ratios for NO release from each component of the MPAN were identified [23,33]. Further immobilization of pDMD resulted in a minor reduction in the NO release rate from 5.6 ± 0.4 × 10^−10^ to 4.1 ± 0.3 × 10^−10^ mol cm^−2^ min^−1^. The decrease in NO generation may be attributed to the loss of Cu^2+^ during pDMD grafting or the shielding effect of the immobilized pDMD (Fig. S5 and Fig. 2H). Free copper ions possessed the capability to penetrate and disrupt bacterial cell membranes, leading to cytoplasmic leakage and ultimately culminating in bacterial death [34]. Consequently, we measured the release of copper ions, and after soaking in phosphate-buffered solution (PBS) for 30 d, the proportion of copper ions retained was 32% (Fig. 2I). Taken together, in this study, we successfully developed a bioinspired zwitterionic block polymer-decorated NO-generating coating armor to modify the surface of CVCs, with the goal of enhancing both anticoagulation and antifouling properties.

### Antibacterial property

**Fig. 3.**
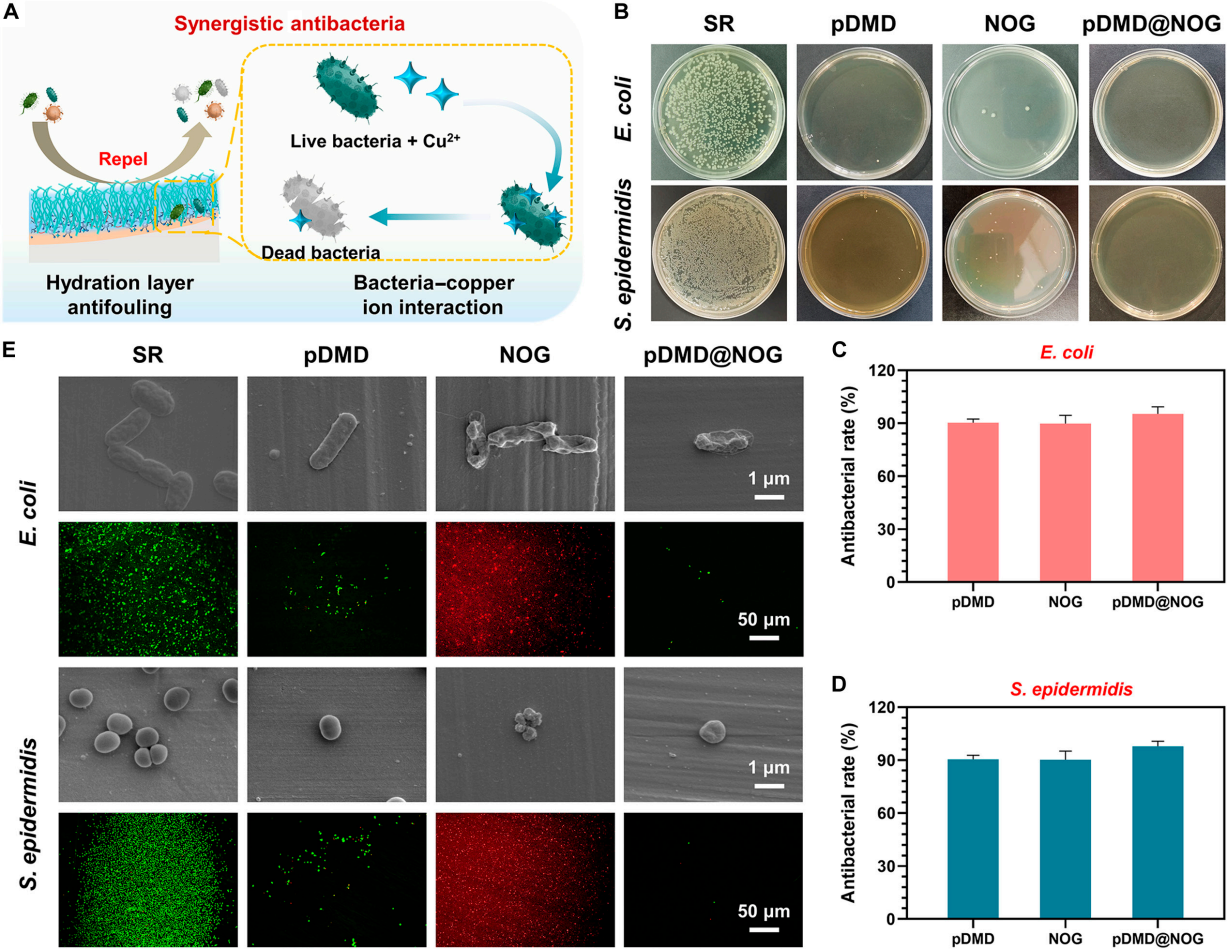
Antibacterial property. (A) Schematic diagram of active attack bacterial of copper ions and passive defense antifouling of hydrated layer formed by zwitterionic charges. (B) Images of *E. coli* and *S. epidermidis* colonies formed on the lysogeny broth agar plate. Antibacterial rates of the sample against (C) *E. coli* and (D) *S. epidermidis* was quantified by counting the number of colonies. (E) SEM images and dead/live staining of *E. coli* or *S. epidermidis* adhered on the bare and modified SR substrates. Statistical analysis was performed using one-way ANOVA with ***P < 0.001.

Microbial adherence to the surface of flexible CVCs is a crucial step in biofilm formation, which substantially contributed to catheter-related infections [35]. Given the bacteriostatic nature of the hydration layer formed around the zwitterionic charges in MPC that could inhibit bacterial adhesion, as well as the sterilization effect of Cu^2+^, we assessed the synergistic antimicrobial properties of pDMD@NOG armor on the surface of CVCs (Fig. 3A). In this study, *Escherichia coli* (*E. coli*) and *Staphylococcus epidermidis* (*S. epidermidis*) were selected for antimicrobial testing due to their frequent association with postinvasive procedure infections [36,37]. Figure 3B clearly demonstrates that pDMD@NOG armor inhibited colony formation of *E. coli* and *S. epidermidis* on agar plates, in contrast to the pristine SR surface. Quantitative analysis further confirmed these observations, revealing that pDMD@NOG armor achieved antimicrobial rates of 99.5% for *E. coli* and 99.3% for *S. epidermidis* (Fig. 3C and D). SEM and bacterial live/dead staining analysis corroborated that pDMD@NOG armor antimicrobial effect resulted from combination of passive defense and active attack mechanisms. Obvious proliferation of *E. coli* and *S. epidermidis* was noted on the SR surface (Fig. 3E). While pDMD and NOG coatings have shown effectiveness in resisting microbial adhesion and proliferation, they also exhibit certain limitations. Passive fouling resistance (pDMD) was observed with a small number of bacteria remaining on the surface, while active attack (NOG) resulted in the accumulation of dead microorganisms. In contrast, bacteria cultured on the pDMD@NOG armor matrix exhibited ruptured bacterial membranes, and almost no microbial adhesion was observed on the material surface. These results indicated that the pDMD@NOG armor provided substantial antimicrobial and antifouling properties to the substrate. The bactericidal and antifouling characteristics of pDMD@NOG armor was ascribed to 2 typical mechanisms: (a) the interaction between Cu^2+^ and microbial membranes, leading to membrane destabilization and increased permeability [34] and (b) the formation of a hydration layer on the surface by zwitterionic block polymer, enhancing surface hydrophilicity and reducing bacterial adhesion. Furthermore, the hydrophilic surface fostered a rinsing effect, further diminishing bacterial settlement on the surface [30]. However, excessive Cu^2+^ release could potentially lead to cytotoxic effects. Therefore, we evaluated the cytocompatibility of pDMD@NOG armor with L929 mouse fibroblast cells, following the guidelines of the American Society for Testing Materials F813 standard. The Cell Counting Kit-8 assay (Fig. S6) showed that pDMD@NOG was neither cytotoxic nor did it hinder the growth and proliferation of L929 cells, demonstrating its superior cytocompatibility.

### Applications in catheters against thrombosis

**Fig. 4.**
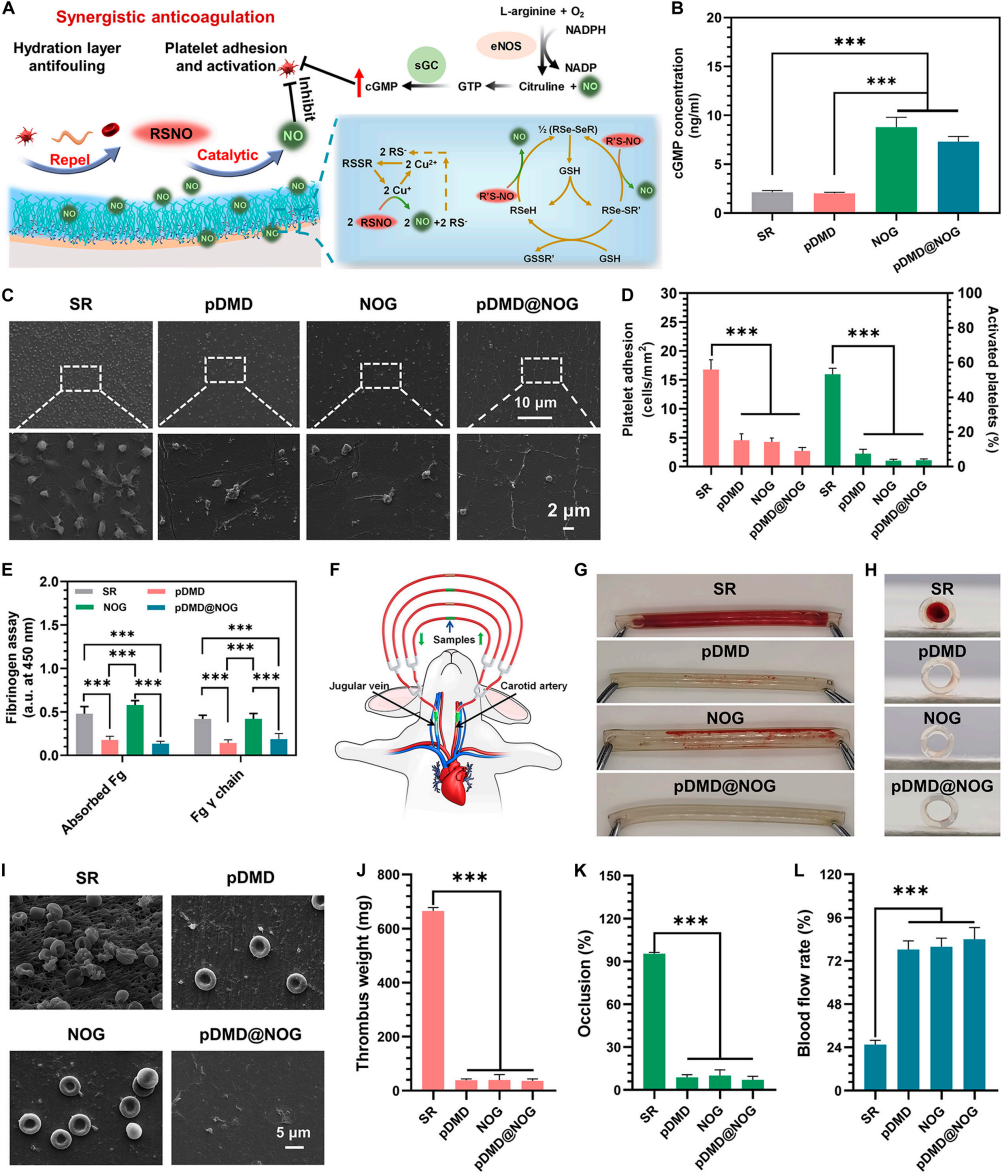
Evaluation of antithrombogenic properties through ex vivo and in vitro blood compatibility tests. (A) Schematic illustration of active anticoagulation of NO and passive antifouling of hydrated layer formed by zwitterionic charges. (B) cGMP expression of platelets. (C) SEM images of platelet adhesion on SR, pDMD, NOG, and pDMD@NOG surfaces. (D) The quantity and activation rate of adherent platelets are determined through counting and the GMP-140 assay. (E) Adsorbed and activated Fg on SR, pDMD, NOG, and pDMD@NOG surfaces. (F) Schematic representation of rabbit carotid arteriovenous shunt model. Optical photographs of side (G) and cross-sectional (H) view of the bare and modified SR catheters after blood circulation. (I) SEM images of the lumen surface morphology. Quantitative results of (J) thrombus weight, (K) occlusion rate, and (L) blood flow rate in different groups. Statistical analysis was performed using one-way ANOVA with ***P < 0.001.

Catheter-related thrombosis represents a substantial clinical challenge for CVCs. Devices implanted in contact with blood may initiate various adverse reactions, including triggering the coagulation cascade and activating the complement system [6]. Among these, platelet and fibrinogen (Fg) adhesion and activation are the key factors causing thrombus formation. Fg is cleaved into fibrin, resulting in the formation of a polymerized fibrin network that serves as the fundamental structure of a blood clot. The clot is intertwined with platelet aggregates, which can be activated through direct contact with the material surface or surface-bound proteins [38]. To address this, we propose combining the fouling-resistant properties of zwitterionic polymer pDMD with the antiplatelet adhesion and activation properties of NO to create a more effective antithrombotic catheter (Fig. 4A). NO, released by endothelial cells, stimulates its primary receptor, soluble guanylate cyclase, leading to platelet cGMP formation. This process reduces intracellular Ca^2+^ levels, inhibiting platelet activation [24]. In our experiments, both NOG and pDMD@NOG significantly increased cGMP expression, effectively inhibiting platelet adhesion and activation (Fig. 4B and C). The pDMD coating also contributed to reduced platelet adhesion due to its protective hydration layer [28]. Quantitative result from Fig. 4D demonstrated that the pDMD@NOG armor had significantly fewer adhered and activated platelets compared to NOG and pDMD coatings, indicating a synergistic effect of NO and MPC in inhibiting platelet adhesion and activation. Similarly, the adhesion and conformational changes of Fg are critical events in thrombus formation. Fg adherence to biomaterial surfaces can initiate inflammation and result in fibrous capsule formation, leading to biomaterial failure or functional loss. Additionally, the γ chain of Fg greatly promoted platelet aggregation, especially through its interaction with the carboxyl-terminal GPIIb-IIIa receptor [39]. As shown in Fig. 4E, in contrast to SR, NOG coating did not cause significant changes in Fg adsorption or conformation. This indicated that NO may not have a direct effect on Fg. In contrast, pDMD and pDMD@NOG significantly reduced the adsorption and conformational changes of Fg. Notably, pDMD and pDMD@NOG exhibited no significant differences in Fg adsorption and conformational changes. It has been reported that hydrophilic surfaces effectively resisted the adhesion of proteins and maintained protein conformational integrity [40]. Subsequently, we demonstrated that the newly synthetic pDMD@NOG material exhibited a safe hemolysis rate of less than 1% (Fig. S7).

To further explore the anticoagulation capabilities of pDMD@NOG catheters, we conducted ex vivo perfusion experiments. Commercially available SR catheters, both unmodified and modified with NOG, pDMD, and pDMD@NOG, were integrated into a rabbit arteriovenous shunt system (Fig. 4F). Following 2 h of flow, the unmodified SR catheter was nearly occluded, whereas the SR catheters modified with pDMD or NOG showed minimal thrombus adhesion. In contrast, the SR catheter coated with pDMD@NOG exhibited almost no apparent thrombus (Fig. 4G and H). SEM analysis revealed the formation of typical thrombi on the exposed SR catheter, comprising platelets and erythrocytes embedded in a fibrin network. On the pDMD- and NOG-modified surfaces, a sparse presence of platelets and erythrocytes was observed within a fine fibrin network. Notably, the pDMD@NOG-modified catheter showed minimal visible components, such as platelets and erythrocytes (Fig. 4I). Thrombus weight measurements further confirmed a significant reduction in thrombus formation on pDMD- or NOG-coated surfaces compared to the bare SR catheter, with the lowest thrombus weight observed on the pDMD@NOG-modified catheter (Fig. 4J). The weight of the thrombus on the catheter showed a direct correlation with the occlusion rate (Fig. 4K) and was inversely proportional to the blood flow rate through the shunt (Fig. 4L). These findings consistently revealed that the pDMD@NOG group exhibited superior antithrombotic efficacy in vitro. Consequently, for further investigation, the SR and pDMD@NOG groups were selected for further investigation in subsequent experiments.

**Fig. 5.**
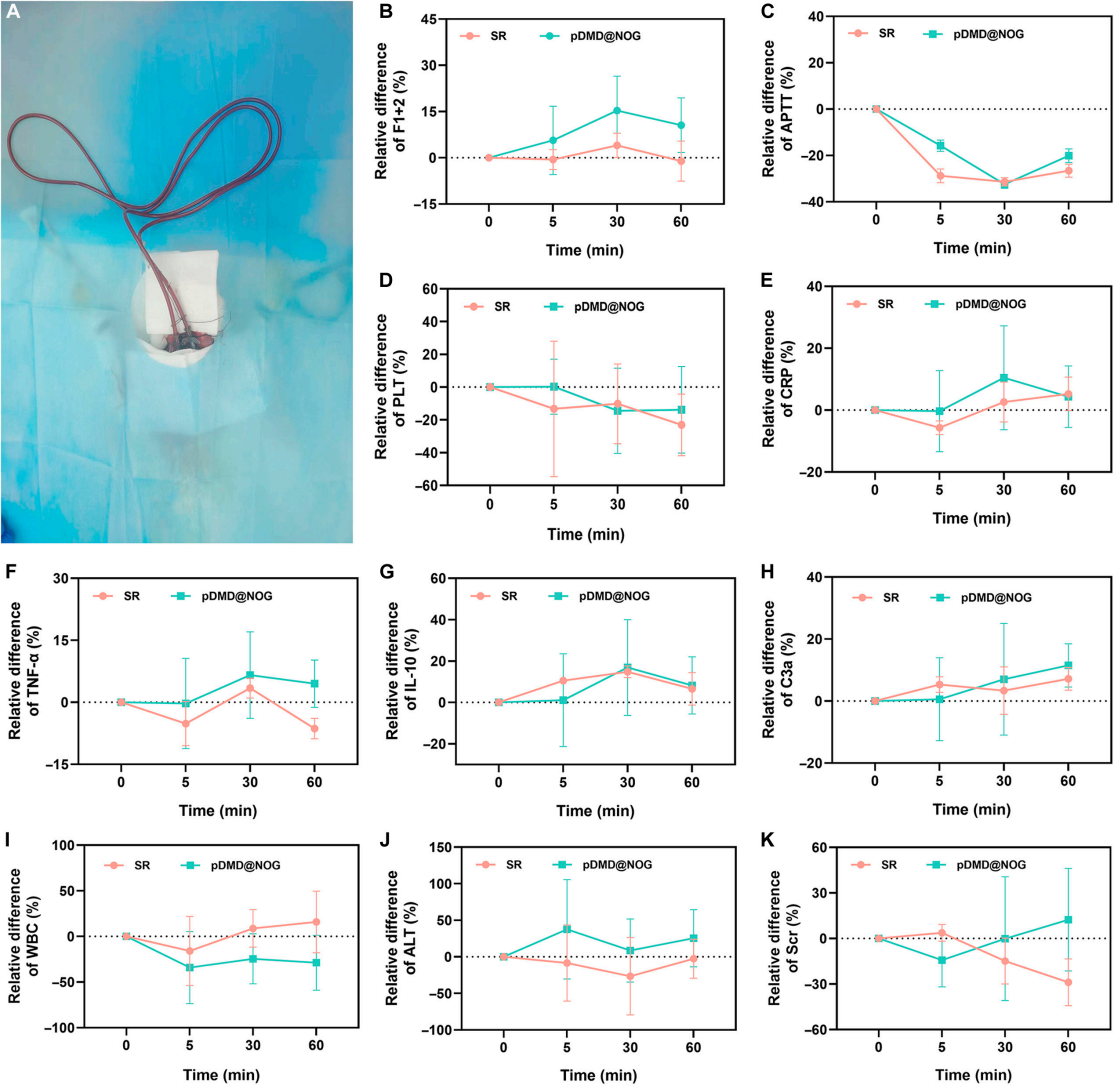
Blood routine and blood biochemistry analysis by in vitro blood circulation test. Coagulation: (A) Rabbit circulatory model used for blood analysis. (B) F1+2 (a general marker for prothrombin activation). (C) APTT. Blood routine: (D) The number of platelets (PLT), (E) CRP, (F) TNF-α, (G) IL-10, (H) C3a (a C3 cleavage fragment), and (I) white blood cells (WBC). Blood biochemistry: (J) The liver enzyme ALT and (K) the kidney parameter Scr. Data are presented as mean ± SD (*n* = 4).

In order to ensure the safety of medical devices that come into prolonged or extensive contact with blood, it is important to assess not only their antithrombotic and antibacterial efficacy but also their impact on the blood, immune system, and other vital organs [41]. In the present study, we placed the 1.6-m-long unmodified or pDMD@NOG-modified SR catheter into the jugular arteriovenous shunt of a rabbit (Fig. 5A). Blood samples were collected at different time intervals (5, 30, and 60 min) for physiological and biochemical assessments, including coagulation, inflammatory response, and organ function (e.g., kidney and liver). Initially, coagulation performance was assessed. The control group showed a higher trend in blood coagulation after prolonged blood contact, as indicated by elevated levels of F1+2 (a general marker of prothrombin activation) (Fig. 5B). However, the activated partial thromboplastin time (APTT) significantly decreased across all groups, possibly attributed to insufficient catalytic NO release (Fig. 5C). Additionally, neither the unmodified nor pDMD@NOG-modified SR catheters had a significant effect on platelet count. (Fig. 5D). Upon blood contact, the catheter was immediately recognized by the immune system, triggering an inflammatory response. After 60 min of circulation, no significant changes in proinflammatory parameters were observed in all groups, including C-reactive protein (CRP, a plasma acute-phase protein used as a measure of acute inflammation; Fig. 5E), tumor necrosis factor-α (TNF-α, a major acute-phase inflammatory cytokine; Fig. 5F), interleukin-10 (IL-10) (a recognized inflammatory and immunosuppressive agent; Fig. 5G), C3a (a C3 fragment indicating activation of the classical or alternative complement pathway; Fig. 5H), and white blood cells (Fig. 5I). These results demonstrated that the proinflammatory indicators in the pDMD@NOG group were consistent with the clinically used unmodified SR group, indicating that the coating did not further promote material inflammatory responses. To assess potential organ and tissue toxicity of the material and coating, we measured blood concentrations of alanine aminotransferase (ALT, a liver enzyme) and serum creatinine (Scr, a kidney parameter). As shown in Fig. 5J and K, both SR and pDMD@NOG surfaces showed no toxicity to organs and tissues during the circulation process, with no significant differences, confirming their excellent biocompatibility.

### Durability of antifouling properties of pDMD@NOG-armored surfaces

**Fig. 6.**
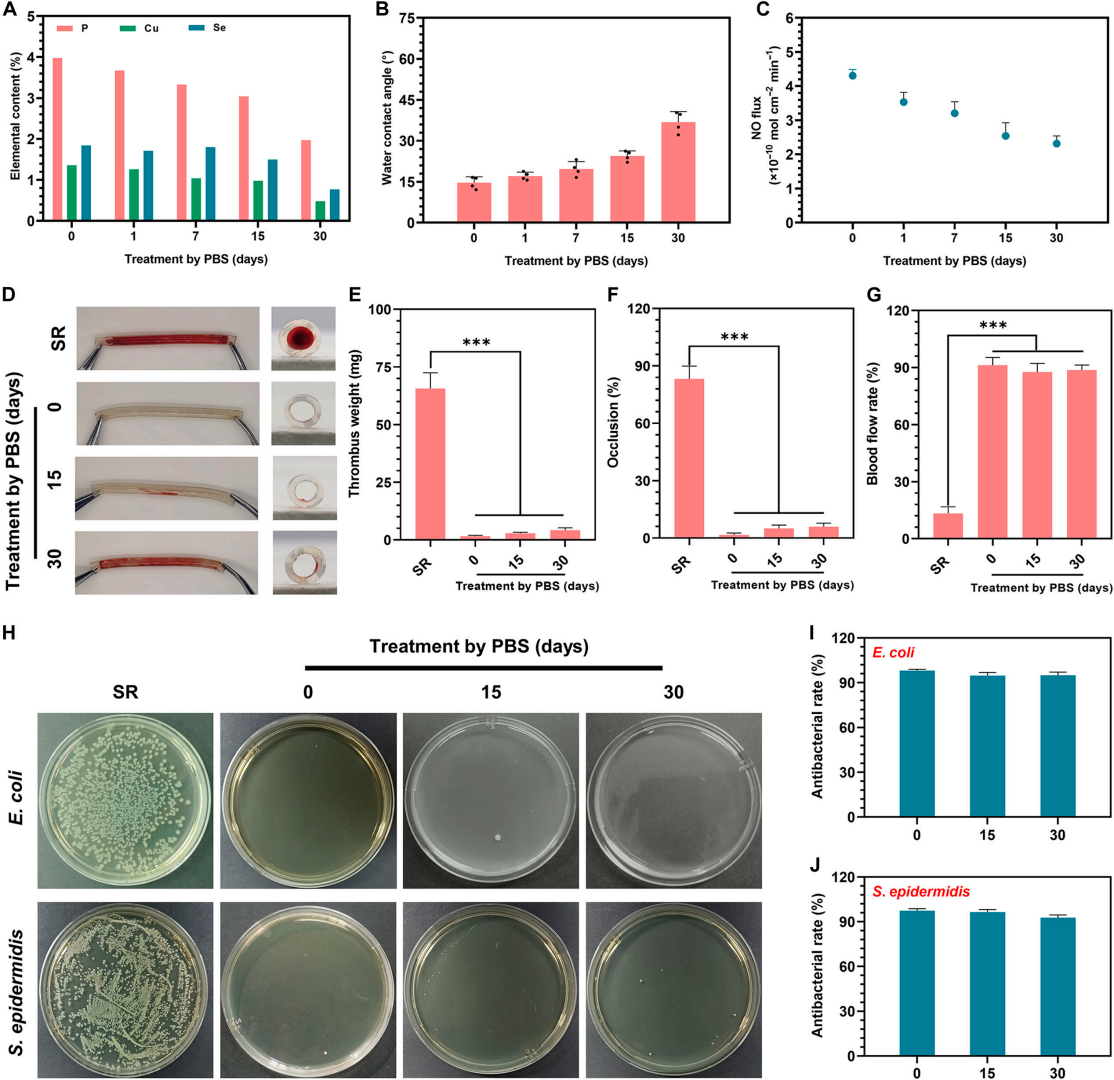
Durability of anticoagulant and antibacterial properties of pDMD@NOG surface. (A) Surface chemical composition, (B) water contact angel, and (C) real-time NO flux of pDMD@NOG coatings after varying immersion times. (D) Images and cross-sectional photographs of the bare or pDMD@NOG-modified SR. (E) Thrombus weight, (F) occlusion rate, and (G) blood flow rate in different groups. (H) Representative colonization of *E. coli* and *S. epidermidis* on bare and pDMD@NOG-modified SR, before and after PBS treatments over various days. Antibacterial rates of (I) *E. coli* and (J) *S. epidermidis* calculated from the results of (H). Statistical analysis was performed using one-way ANOVA with ***P < 0.001.

Given the potential for long-term implantation of the pDMD@NOG armor on CVCs, its antibacterial and antithrombotic effects were evaluated after immersion in PBS for varying periods. XPS analysis showed a decrease in the surface content of P, Cu, and Se elements after treatment by PBS for 30 d (Fig. 6A). Similarly, the WCA (Fig. 6B) and NO release capacity (Fig. 6C) demonstrated a positive correlation. After immersion in PBS for 30 d, the WCA increased from the initial 12.1° to approximately 35.4°, and the NO release capacity decreased by 52%, with a release rate of 2.1 ± 0.4 × 10^−10^ mol cm^−2^ min^−1^. Yang et al. [42] demonstrated that the choline phosphate coating retained substantial antifouling capabilities even when the WCA value was approximately 41°. Similarly, the release of NO continued to be effective in preventing platelet adhesion within a specific range [43]. These results indicated that the pDMD@NOG surface maintained both passive defence and active attack capabilities, even after 30 d in PBS.

To confirm the above results, an ex vivo circulation experiment was conducted to evaluate the durability of the antithrombotic properties of pDMD@NOG-modified tubes after exposure to PBS treatments for varying durations (15 and 30 d). Following 2 h of circulation, the unmodified SR tube was almost completely occluded (Fig. 6D); in contrast, the pDMD@NOG-modified tube treated with PBS for 30 d demonstrated minimal thrombus adhesion and slight occlusion (Fig. 6E). The thrombus formation could be attributed to the reduced NO relase, diminishing its efftiveness against platelet adhesion and activation. However, owing to the presence of zwitterionic charges present on its surface, the tube maintained its hydrophilicity, ensuring that the surface of the catheter remained actively antiadhesive. Quantitative analysis, as shown in Fig. 6E, indicated a significant reduction in thrombus weight on the pDMD@NOG-armored surface from 380.2 ± 20.7 mg on the bare SR surface to 2.5 ± 0.4 and 8.6 ± 6.4 mg after PBS treatment for 15 and 30 d, respectively. The occlusion rate (Fig. 6F) and blood flow rate (Fig. 6G) on the pDMD@NOG-armored surface remained almost constant after 30 d of PBS treatment. Antibacterial tests revealed that even after 30 d of continuous PBS treatment (Fig. 6H), the pDMD@NOG-armored surface inhibited 98.5% of *E. coli* (Fig. 6I) and 97.6% of *S. epidermidis* growth (Fig. 6J). The excellent antibacterial performance could be attributed to the antifouling properties of pDMD on the treated surface and the retained Cu ions. These results suggest that the pDMD@NOG armor holds promise for long-term applications in blood-contacting devices or implants.

### In vivo anti-infective activity and anticoagulant performance of the pDMD@NOG-armored CVCs

**Fig. 7.**
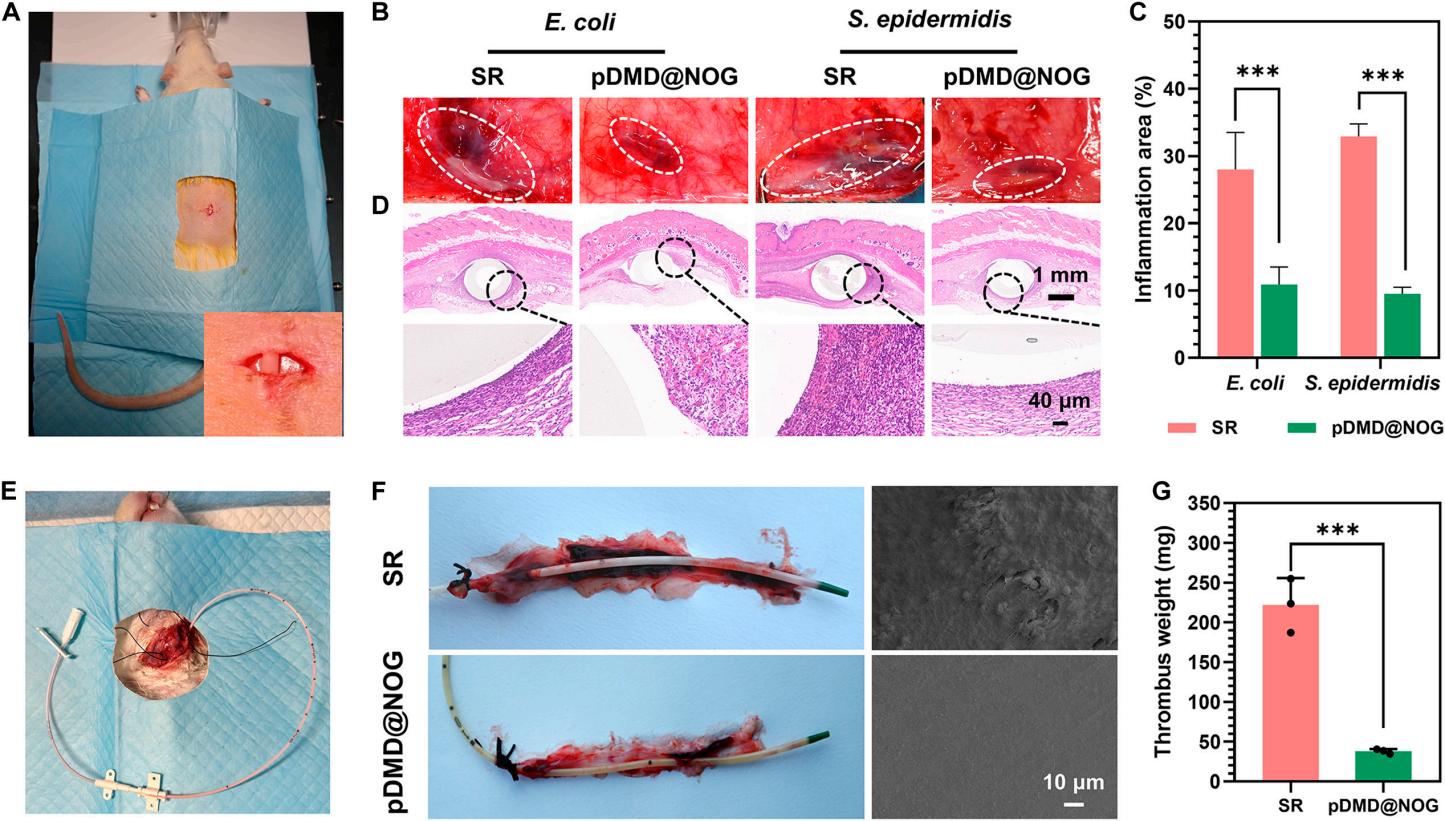
In vivo infection resistance activity and anticoagulant performance of the pDMD@NOG-armored CVCs. (A) Schematic diagram of subcutaneous infection model in rats. (B) Images depicting inflammation associated with SR and pDMD@NOG-modified samples after 7 d of animal experimentation. (C) Inflammation area statistics for the bare and pDMD@NOG-modified samples. (D) Histological staining (H&E staining) showed tissue sections around the bare and pDMD@NOG-modified samples. (E) Diagram of rabbit venous anticoagulation model. (F) Evaluation of CVCs samples in a rabbit model. Images of the catheter and associated blood vessels were captured. (G) Thrombus weight statistics after 8 h of venous placement. Statistical analysis was performed using one-way ANOVA with ***P < 0.001.

In vitro and ex vivo, the bioinspired pDMD@NOG coating demonstrated excellent antibacterial and anticoagulant properties. However, for in vivo environment, involving complex physiological processes such as blood flow homeostasis and inflammation, it was necessary to further evaluate its antibacterial and anticoagulant activity. Initially, we exposed unmodified and pDMD@NOG-modified CVCs to *E. coli* and *S. epidermidis* in vitro (Fig. 7A), and these exposed samples were implanted subcutaneously in healthy rats and maintained for 7 d. It was observed that the bare CVCs showed marked inflammation, while the pDMD@NOG-modified CVCs showed minimal signs of inflammation (Fig. 7B). Further quantitative analysis indicated that the pDMD@NOG-modified CVCs reduced the area of inflammation by 64.5% and 70.3% for *E. coli* and *S. epidermidis*, respectively, compared to the unmodified CVCs (Fig. 7C). Hematoxylin and eosin (H&E) staining results further indicated a higher presence of inflammatory cells (blue color) in tissues surrounding the uncoated CVCs compared to the pDMD@NOG-modified CVCs, implying a significant reduction in bacterial counts and inflammatory cell numbers in live animals (Fig. 7D). Collectively, these results indicated that pDMD@NOG armor maintained its excellent anti-infective activity in vivo*.*

To assess the in vivo thrombogenic performance of the pDMD@NOG-modified CVCs, we utilized a rabbit venous implantation model (Fig. 7E). In the absence of any anticoagulant, we implanted pDMD@NOG-modified and commercial CVCs into different rabbit jugular veins and directly compared their performance. After 8 h of implantation, pronounced thrombus accumulation was observed on the commercial CVCs, while the pDMD@NOG-modified CVCs displayed minimal thrombus adhesion to the catheter or adjacent vessel walls (Fig. 7F, left). Additionally, SEM results revealed pronounced thrombi on the surface of commercial CVCs, comprising Fg networks and blood cells, while the pDMD@NOG-modified sample surface exhibited negligible platelet and blood cell adhesion (Fig. 7F, right). Quantitative results showed a 90% reduction in thrombus associated with the pDMD@NOG-modified CVCs (Fig. 7G). These results conclusively demonstrated that pDMD@NOG maintained its excellent anticoagulant properties in vivo.

## Discussion

In recent decades, researchers have devoted efforts to enhancing the biocompatibility of CVCs in order to address the prevalent issues of thrombosis and infection after implantation [8,9]. Strategies that combine passive defense (e.g., immobilization protein-resistant polymers) and active attack (e.g., integration of bioactive molecules) have gained substantial attention due to their excellent anticoagulant and antibacterial properties. In this context, our team proposed a surface engineering strategy based on zwitterionic polymers and amyloid-like proteins to enhance the anticoagulant and anti-infective properties of CVCs [44]. Although the current modified catheters demonstrated antibacterial effects for up to 30 d, their anticoagulant performance was limited to only 15 d due to the lack of active anticoagulant capabilities. This discovery emphasizes the necessity of integrating both antibacterial and anticoagulant functionalities in a combined strategy to achieve long-lasting effects.

In our study, we present the pDMD@NOG coating, which can be applied to commercial medical-grade CVCs to create surfaces that are resistant to thrombosis and biofouling. This coating provided a durable synergistic effect, effectively combating Fg and platelet adhesion, while also exhibiting antibacterial properties both in vitro and in vivo. Notably, the coating retained excellent antibacterial and anticoagulant capabilities even after exposure to PBS treatment for up to 30 d, overcoming the constraints of prior research.

Considering the potential application of the pDMD@NOG coating in medical products, the stability of the coating during storage is a crucial factor. To assess the storage performance of the coating, we monitored the WCA and NO release rate. Figure S8 reveals that after storing the pDMD@NOG-modified SR in sterile bags at room temperature for 21 d, there was almost no change in both the WCA and NO release rate. These results indicate that the coating has excellent storage potential. Furthermore, all medical devices must undergo sterilization before use on humans, making it essential for the coating to retain its functionality after sterilization. In this study, we employed the commonly employed laboratory UV sterilization method to evaluate the stability of the coating. The experiment confirmed that even after sterilization, the coating surface effectively maintained its hydrophilicity and NO release capability (Fig. S8). This positive outcome is attributed to the mussel-inspired adhesive properties of DA and the covalent coupling process that securely attaches the polymer to the catheter surface.

Despite the considerable advantages of our strategy, there are certain limitations that need to be addressed. A primary concern is the deep brown color of the coating attributed to its polyphenol content. This color can impede the visibility of blood in surgical environments, potentially complicating real-time assessment of blood flow. Additionally, while we validated the antibacterial and anticoagulant properties of the coating before and after UV sterilization under laboratory conditions, it is necessary to further verify these results using clinical sterilization methods (e.g., steam sterilization [autoclaving] and ethylene oxide fumigation) to ensure reproducibility and stability. Future research efforts should focus on overcoming these limitations to improve the applicability of the material in surgical environments and ensure its reliability and safety in clinical applications.

In summary, we successfully developed a bioinspired zwitterionic block polymer-decorated NO-generating coating armor to modify the surface of CVCs. The pDMD endowed the CVCs with superior hydrophilicity (~12.1°) and a low friction coefficient (0.03). The Cu-DA/SeCA network promoted NO release (4.1 ± 0.3 × 10^−10^ mol cm^−2^ min^−1^), maintaining hydrophilicity (~35.4°) and effective NO release (2.1 ± 0.4 × 10^−10^ mol cm^−2^ min^−1^) even after 30 d of immersion in PBS. Through the synergistic interaction of regional defense (pDMD) and active attack (NO/Cu^2+^), our surface functionalization strategy effectively combated in vitro adhesion of bacteria, Fg, and platelets. In practical applications, this strategy showed potential promise for intravascular catheters, exhibiting outstanding antithrombogenic and antimicrobial capabilities even after 30 d of immersion in PBS, as evidenced by ex vivo blood circulation tests. More importantly, the pDMD@NOG-armored catheter effectively prevented subcutaneous bacterial-induced infections and sustains robust anticoagulant functions for up to 8 h without additional anticoagulants. We anticipated that this facile technique could be easily applied to coat most biomaterial surface, preventing infections associated with various cardiovascular biomaterials and interventional/implantable devices.

## Materials and Methods

### Materials and reagents

Bovine serum albumin, methacrylic anhydride, and DA hydrochloride were obtained from J&K Scientific Ltd., Beijing, China. MPC (98%) was purchased from Joy-Nature Institute of Technology, Nanjing, China. Sodium bicarbonate (NaHCO_3_), sodium tetraborate (NaB_4_O_7_), and azobiisobutyronitrile (AIBN) were sourced from Aladdin Bio-Chem Technology Co., Ltd., Shanghai, China. CuCl_2_·2H_2_O, sodium hydroxide (NaOH), bacterial culture medium S-nitroso-N-acetyl-DL-penicillamine (SNAP), selenocysteamine (SeCA), and L-glutathione (GSH) were purchased from Sigma-Aldrich, Shanghai, China. High-purity titanium alloy (Ti6Al4V) sheet was obtained from Goodfellow Cambridge Ltd., Huntingdon, UK. Anti-Fg gamma chain antibody, mouse anti-human Fg/horseradish peroxidase (HRP), and goat anti-mouse immunoglobulin G/HRP were purchased from Beijing Biosynthesis Biotechnology Co., Ltd. Beijing, China.

### Synthesis of pDMD copolymer

DMA was synthesized following the method outlined in our previous study [45]. The p(DMA-*b*-MPC-*b*-MPC) copolymer was prepared through RAFT polymerization technique, employing DMA and MPC monomers with 4,4’-azobis(4-cyanovaleric acid) (ACVA) serving as the initiator. First, DMA (120 mg, 0.6 mmol), 2-cyanoprop-2-yl dithiobenzoate (13 mg, 0.06 mmol), and ACVA (2.8 mg, 0.01 mmol) were dissolved in a 50-ml mixture of N,N-dimethylformamide and deionized water at a volume ratio of 1:1. Under the protection of nitrogen, the reaction mixture was proceeded at 65°C for 24 h and cooled to room temperature. Then, MPC (1.2 g, 4 mmol) and ACVA (2.8 mg, 0.01 mmol) were added into the mixture, reacting at 65 °C for 24 h. Lastly, after cooling the solution to room temperature, DMA (120 mg, 0.6 mmol) and ACVA (2.8 mg, 0.01 mmol) were added, and the process was extended at 65°C for 24 h under nitrogen protection. All the reaction processes were monitored by nuclear magnetism. Subsequently, the reaction mixture underwent dialysis and freeze-drying, yielding the copolymer pDMD.

### Preparation of functional coating armor

NO-generating coating was meticulously prepared on SR substrates and SR catheters using a one-step dip-coating method, following previously established methodologies [33]. The specific procedure was detailed as follows. DA (1 mg/ml), SeCA hydrochloride (1 mg/ml), and a copper (II) chloride dihydrate solution (CuCl_2_·2H_2_O, 20 μg/ml) were dissolved in a tris buffer solution (pH 8.5). This reaction was conducted at 25 °C for 24 h. Then, the surfaces were ultrasonically cleaned to ensure optimal coating adherence, and referred to as the nitric oxide-generating (NOG) coating. The NOG coatings were immersed in a solution of pDMD (16 mg/ml) in tris buffer (pH 8.5) at 37 °C for 24 h. Finally, they were thoroughly rinsed with distilled water to remove residual substances and termed as pDMD@NOG.

### Characterization of pDMD and pDMD@NOG armor

The structure of pDMD was verified through ^1^H NMR spectroscopy using an AVANCE III HD 400-MHz NMR spectrometer (Bruker, Switzerland), with deuterium oxide (D_2_O) serving as the solvent. The weight-average molecular weight of the pDMD copolymer was determined using GPC (Malvern Instruments, UK), employing sodium nitrate as the eluent (0.1 M, flow rate 0.7 ml/min). The COF on the surface of the materials was assessed using an atomic force microscope (MFP-3D, Asylum Research, Santa Barbara, USA). The thickness of the coating was measured using a spectroscopic ellipsometer (AutoRetarderTM, W-VASE, J.A. Woollam, USA), calculating its ψ (± 0.015°) and Δ (± 0.08°) values across a wavelength range of 240 to 1,100 nm. The WCA was evaluated using a Krüss GmbH DSA 100 Mk 2 goniometer (Hamburg, Germany). Surface morphology of both uncoated and coated samples was examined using SEM (JSM-7800F, JEOL Ltd., Japan) after procedures of dehydration, dealcoholization, and drying. The chemical structures and components of the coating were analyzed by grazing angle attenuated total reflection FTIR spectroscopy (NICOLET 5700) and XPS (AXIS Supra, Kratos Analytical Inc., Japan). Characteristic peaks of polymers and DA were identified using a UV spectrophotometer (TU-1901, Beijing Puxi General Instrument Co., Ltd., China).

### Lubrication property

We evaluated the lubrication properties of pDMD copolymers using an atomic force microscope in contact mode. Before conducting the experiments, we precisely calibrated the cantilever’s spring constant and determined its lateral sensitivity. The COF between PS microspheres and SR samples, both uncoated and coated, was precisely measured under ambient conditions in a PBS. The experiments were carried out with applied loads ranging from 50 to 250 nN, resulting in contact pressures of 27.3, 39.4, and 46.7 MPa for loads of 50, 150, and 250 nN, respectively. The experimental protocol also involved modifying the scan rates between 1 to 3 Hz while maintaining a constant sliding distance of 20 μm. To ensure data reliability and consistency, each experiment was replicated on 3 distinct 20 μm × 20 μm rectangular sections, selected randomly and subjected to the same experimental conditions. The mean COF and its SD were derived from 3 distinct measurements, each based on 256 scans performed on the selected area.

### Nitric oxide catalytic release test

Real-time NO release rates of NOG and pDMD@NOG coatings were measured using nitric oxide analyzer (Seivers 280i, Boulder, CO). For this analysis, samples were immersed in the reaction buffer solution composed of 5 ml of PBS (pH 7.2), 10 μM SNAP, and 10 μM GSH. All the measurements were conducted at a temperature of 37 °C in a controlled environment shielded from light sources. The real-time NO levels were recorded at quarter-second intervals following the insertion of the coated samples into the reaction buffer solution. Data acquisition began following an 8-min baseline standardization period. After reacting for 40 min, the samples were removed and the data was collected. The flux of NO was determined utilizing a calibration curve, with details discussed elsewhere [46].

### Sample sterilization

All experimental samples underwent UV sterilization before any in vitro and in vivo biological experiments. The sterilization process involved placing the prepared samples on a biosafety cabinet and subjecting them to UV irradiation for 30 min. Following this, the samples were sealed in sterile sample bags for subsequent use.

### Antibacterial property

The antibacterial properties of the samples were measured according to ISO22196-2011. The selected strains included gram-positive *S. epidermidis* (strain ATCC 12228) and gram-negative *E. coli* (strain ATCC 25922). Monoclonal bacteria were cultured in nutrient broth for 12 h, and UV spectroscopy was employed to determine bacterial concentration as 5 × 10^5^ CFU ml^−1^. Subsequently, 0.1 ml of the bacterial solution was added to both the presterilized experimental and control samples and incubated them at 37°C for 24 h. Afterwards, 1 ml of physiological saline was added to each sample to disperse the bacteria evenly. A 20-μl aliquot of the bacterial suspension was then spread evenly on an agar plate. Following a further 24-h incubation at 37°C, photographs were obtained using an optical microscope.

Formula 1 was used to calculate the antibacterial rate (*R*), where *NC* represents the number of colonies on the control sample, and *N* denotes the number of colonies on the coated sample.

$$R=\left(\mathrm{NC}-N\right)/\mathrm{NC}\times 100\%$$ (1)

To more closely investigate the antibacterial mechanism, we conducted SEM analyses and live/dead bacterial staining experiments. Following the previously outlined procedures, bacteria were inoculated and incubated on the surfaces of the samples for a period of 24 h. After rinsing the sample with physiological saline, a dye mixture of SYTO9 and propidium iodide was applied for staining for 15 min, followed by observation under a fluorescence microscope. Similarly, the samples were processed by fixation with glutaraldehyde, followed by dehydration and dealcoholization treatments, prior to observation with the SEM.

### Platelet adhesion and activation and expression level of cGMP

Fresh human blood was obtained from the Blood Collection Centre of the Tenth Affiliated Hospital of Southern Medical University, Dongguan, China, with 3.8% sodium citrate used as an anticoagulant. The blood was centrifuged at various speeds for 15 min to obtain platelet-rich plasma (PRP, 1,500 rpm) and platelet-poor plasma (3,000 rpm) for this study, with the experiments conducted within 12 h.

Initially, 500 μl of PRP was combined with 20 μl of NO donor solution (SNAP [10 μM] and GSH [10 μM]) and dropped onto the sample surface for 2 h at 37 °C. Nonadherent platelets were washed off with PBS. Then, the sample was fixed in a 2.5% glutaraldehyde solution for 12 h and proceeded with gradient dehydration and dealcoholization. Finally, the platelet morphology was examined using SEM. To determine cGMP expression levels, the samples were incubated in PRP for 30 min, followed by cleaning with saline and sonication with 10% Triton X-100 (0.1 ml) for 5 min. Subsequently, the resulting suspension was centrifuged (2,500 rpm, 15 min), and the supernatant was collected. The cGMP expression in platelets was analyzed using a cGMP enzyme-linked immunosorbent assay (ELISA).

### Fg adsorption and activation

To investigate the interaction between Fg and the sample, the following experimental procedure was used. First, 100 μl of platelet-poor plasma was applied to the sample and incubated at 37°C for 2 h. After incubation, the samples were washed with saline solution and blocked with 5% bovine serum albumin solution for 30 min. For the Fg adsorption assay, after blocking, experiments were conducted according to the instructions provided with the human Fg ELISA kit. Fifty microliters of a mouse anti-human Fg/HRP antibody (1:1,000 dilution) was dropped onto each sample, followed by an incubation at 37 °C for 1 h. Subsequently, 100 μl of tetramethylbenzidine, prepared by mixing Solutions A and B in a 1:1 ratio, was added to each sample. After a 10-min reaction period, the reaction was quenched with 50 μl of 1M H_2_SO_4_. The absorbance was measured at 450 nm using a microplate spectrophotometer. The activation of Fg adsorbed on the sample surfaces was indirectly assessed by measuring the presence of the Fg gamma chain. In a similar procedure, 50 μl of an anti-Fg gamma chain antibody (1:3,000 dilution) was added to each sample surface and incubated at 37°C for 1 h. This step was followed by an incubation with 50 μl of goat anti-mouse immunoglobulin G/HRP at the same temperature and duration. The color development and absorbance measurement steps were identical to those in the initial Fg adsorption assay.

### Cell compatibility

To assess cytocompatibility with L929 mouse fibroblasts, the cells were inoculated onto the substrate at a density of 2 ×10^4^ cells/cm^2^ and cultured in Dulbecco's Modified Eagle Medium/Nutrient Mixture F-12 medium enriched with 20% fetal bovine serum. The samples were incubated in a cell culture incubator for 24 and 72 h. Cell viability was assessed using the Cell Counting Kit-8 assay, following the provided instructions.

### Hemolysis rate evaluation

The erythrocyte compatibility of the pDMD@NOG coating was evaluated using an in vitro hemolysis test. The whole blood was diluted with physiological saline at a 5:4 volume ratio for subsequent use. All samples were immersed in physiological saline at 37°C for 30 min and then incubated in 10 ml of physiological saline mixed with 0.2 ml of diluted blood for 1 h at 37°C. In this test, saline solution (0.9% [w/v] NaCl) served as the negative control and deionized water as the positive control for calculating the degree of hemolysis. A microplate reader was used to quantify the absorbance of hemoglobin liberated from red blood cells after the sample solutions were collected and centrifuged for 5 min at 3,000 rpm.

Hemolysis ratios were calculated using Formula 2, where *A* represents the absorbance value of the sample, and *B* and *C* denote the absorbance values of the positive and negative controls, respectively.

$$\%\text{Hemolysis}=\left[\left(A-C\right)/\left(B-C\right)\right]\times 100$$ (2)

### Ex vivo hemocompatibility test

All animal experiments conducted in this study were adhered to the guidelines of the Council for the Purpose of Control and Supervision of Experiments on Animals, Ministry of Public Health, China. Approval for these experiments was obtained from the Dongguan People’s Hospital Laboratory Animal Welfare and Ethics Committee (Approval No. IACUC-AWEC-202311007).

To assess the anticoagulant capability of the pDMD@NOG surface under blood circulation conditions, in vitro blood circulation assays were performed. Six New Zealand white rabbits, each weighing between 2.5 and 3.5 kg, were utilized, with 4 parallel samples in each group for testing. The bare, NOG-, pDMD-, and pD MD@NOG-coated catheters were connected to a vein–artery extracorporeal circulation circuit (ECC) for experiments. Blood flow through the ECC was monitored in real time for 2 h. Subsequently, the samples were collected, photographed, and weighed, and the blood flow rate was quantified. The samples were fixed in a 2.5% glutaraldehyde solution for 12 h. Following dehydration and dealcoholization, the samples were analyzed with SEM for morphological observations.

### Blood analysis

Eight adult New Zealand white rabbits were employed in the ex vivo blood circulation experiments. SR tubes, both uncoated (bare SR) and coated with pDMD@NOG, were connected into an ECC. To ensure optimal physiological conditions for the animals and to better simulate clinical scenarios, longer tubing measuring 1.6 m in length and 3 mm in diameter was employed, maximizing the contact area and thereby enhancing the experimental effectiveness. Blood samples were collected from the rabbits at 4 intervals: immediately (0 min) and at 5, 30, and 60 min after the initiation of blood flow. Comprehensive analyses of these samples included blood composition assessments (complete blood count and serum parameters) and coagulation status evaluations (APTT). Furthermore, various serum indicators such as CRP, C3a, IL-10, TNF-α, F1+2, CRE, and ALT were quantified using ELISA at both the start and end of the experiments. Detailed methodologies for APTT and ELISA assessments were available in previously published literature [47].

### Stability studies of coatings

The pDMD@NOG-armored substrates or tubes were placed in 5 ml of PBS solution and then stored in a shaker maintained at 37°C for durations of 1, 7, 15, and 30 d. To ensure a consistent environment, the PBS solution was refreshed every 12 h during the soaking period. Following these periods, all samples underwent comprehensive characterization, including XPS, NO analysis, WCA measurements, as well as antibacterial and ex vivo thrombogenicity tests.

### In vivo anti-infection capacity of pDMD@NOG-armored CVCs exposed to bacteria

Initially, unmodified and NOG-modified sterile CVC sections, each measuring 1 cm, were exposed to either *E. coli* or *S. epidermidis* solutions with an approximate concentration of 1×10^7^ CFU/ml. These samples were then incubated at 37°C for 12 h, followed by a transfer to PBS at the same temperature for about 3 h. Subsequently, these bacteria-exposed CVC sections were implanted into the backs of anesthetized Sprague-Dawley rats, with the surgical sites being sutured shut. After a week of observation, signs of inflammation at the implant sites were visually assessed and documented. To conduct a more detailed examination of inflammation, the samples were fixed in paraformaldehyde, sectioned, and then stained with H&E.

### Rabbit in vivo thrombogenicity studies

New Zealand white rabbits (*n* = 3) were anesthetized using sodium pentobarbital. Unmodified and pDMD@NOG-modified CVCs were inserted approximately 10 cm into jugular vein of each animal, with the catheters secured and the incisions closed. Anticoagulants were not used during this period. Approximately 8 h later, heparin (100 U/kg) was administered to the animals to prevent postmortem coagulation. Euthanasia was then performed, and the neck veins containing the catheters were completely removed. The veins were longitudinally opened to inspect for evidence of thrombus formation around the devices and associated blood vessels. Photographs were taken, thrombus length was measured, and thrombus weight was quantified to assess the accumulation of thrombi associated with each sample.

### Statistical analysis

Experimental data were reported as mean ± SD (*n* = 4), with error bars representing this variation in all statistical graphs. The significance of differences between groups was evaluated using a one-way analysis of variance (ANOVA) with IBM statistical software (v.20, SPSS, Chicago, IL, USA), employing consistent methodologies. These in vitro studies were performed across 4 independent experiments, each comprising at least 3 technical replicates.

## Data Availability

The data that support the findings of this study are available from the corresponding author upon reasonable request.

## Supplementary Materials

Supplementary 1 Search strategy Figs. S1 to S8
