# Supplementary material for: Bioinspired Zwitterionic Block Polymer-Armored Nitric Oxide-Generating Coating Combats Thrombosis and Biofouling
Source: Research (Wash D C). 2024 Aug 1;7:0423. doi: 10.34133/research.0423 (PMC11290871; doi:10.34133/research.0423)
Supplement: Supplementary 1 — Search strategy Figs. S1 to S8 [file research.0423.f1.zip › Supplementary Materials.docx]

Supplementary Materials

**Bioinspired Zwitterionic Block Polymer-Armored Nitric Oxide-Generating Coating Combats Thrombosis and Biofouling**

Qing Ma ^1, 2^, Wentai Zhang ^2^, Xiaohui Mou ^1, 2^, Nan Huang ^2, 3^, Haimang Wang ^4, 5 *^, Hongyu Zhang ^4, *^, and Zhilu Yang ^1, 2 *^

^1^ School of Materials Science and Engineering, Key Lab of Advanced Technology of Materials of Education Ministry, Southwest Jiaotong University, Chengdu, 610031, China.

^2^ Dongguan Key Laboratory of Smart Biomaterials and Regenerative Medicine, The Tenth Affiliated Hospital, Southern Medical University, Dongguan, 523059, China.

^3^ GuangZhou Nanchuang Mount Everest Company for Medical Science and Technology, Guangzhou, 510670, China.

^4^ State Key Laboratory of Tribology in Advanced Equipment, Department of Mechanical Engineering, Tsinghua University, Beijing, 100084, China.

^5^ Wenzhou Institute, University of Chinese Academy of Sciences, Wenzhou 352001 Zhejiang, China

^*^Correspondence author; wanghaimag@163.com (H. W.); zhanghyu@tsinghua.edu.cn (H. Z.); zhiluyang1029@smu.edu.cn (Z. Y.)


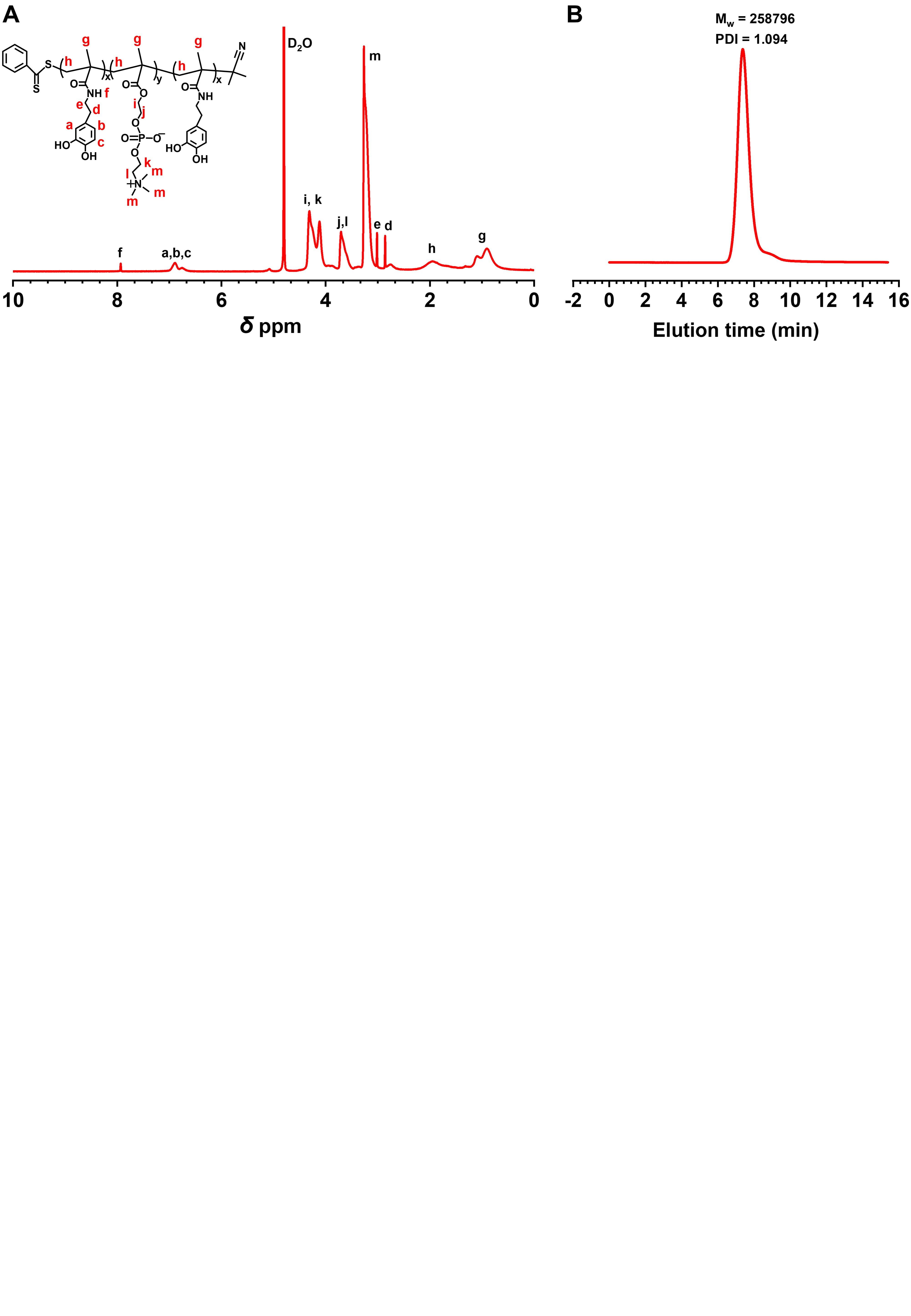


Fig. S1. Characterization of the synthesized pDMD block polymer. (A) ^1^H NMR spectrum and (B) GPC analysis.

**
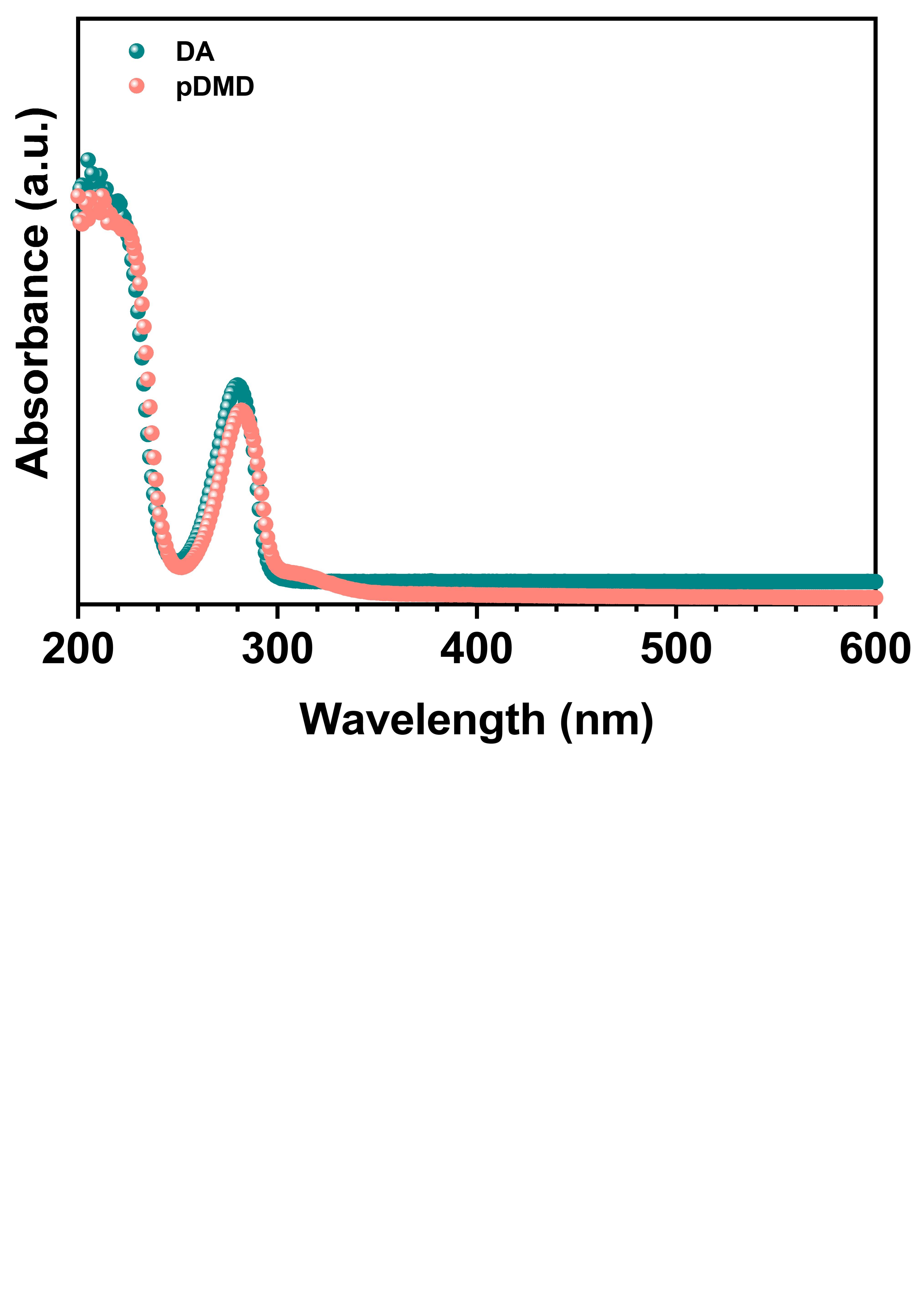
**

Fig. S2. UV−vis spectra for the dopamine (DA) and pDMD solutions at the pH value of 7.4.

**
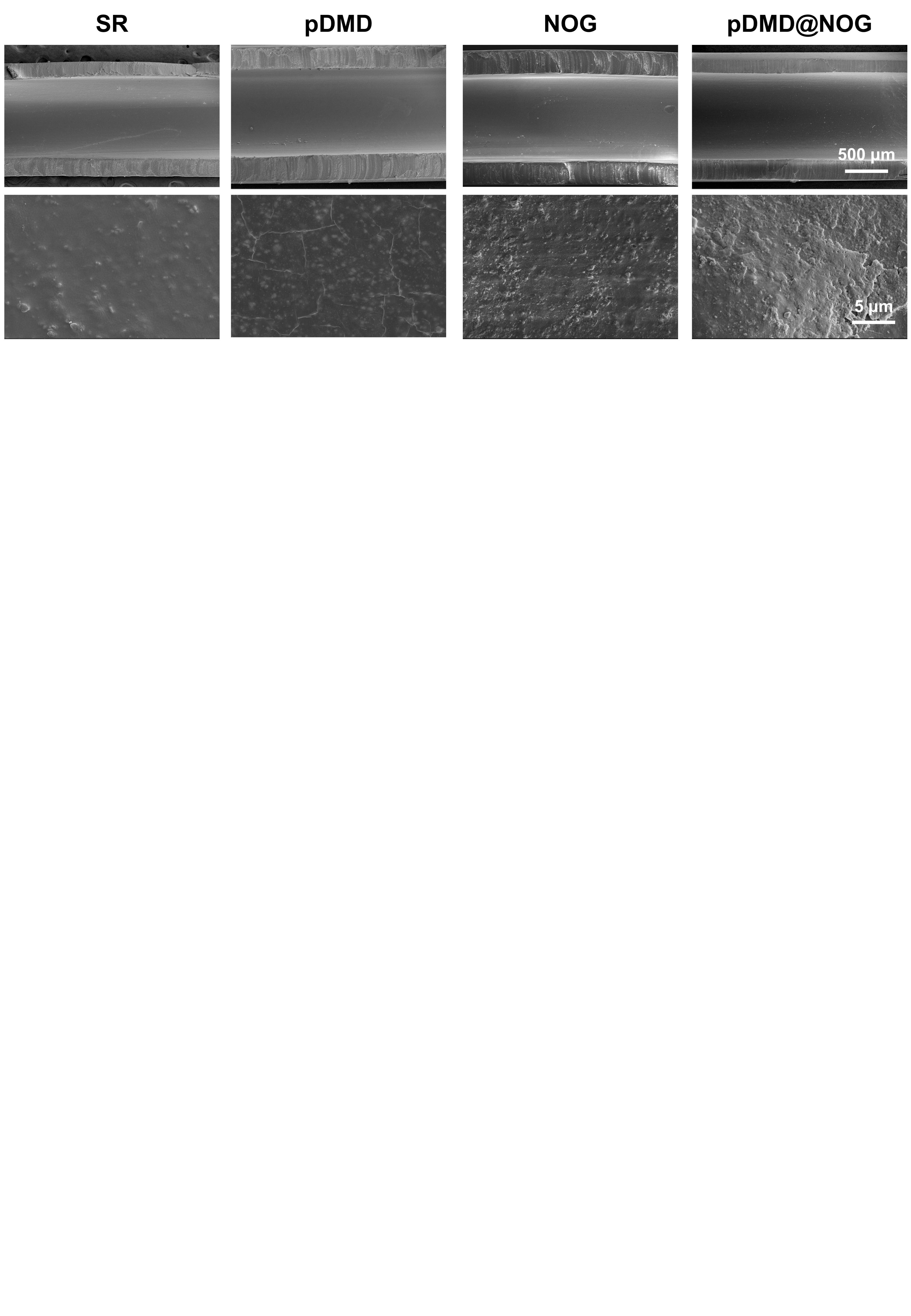
**

Fig. S3. SEM images of the inner surface of different (bare and decorated) catheter samples.

**
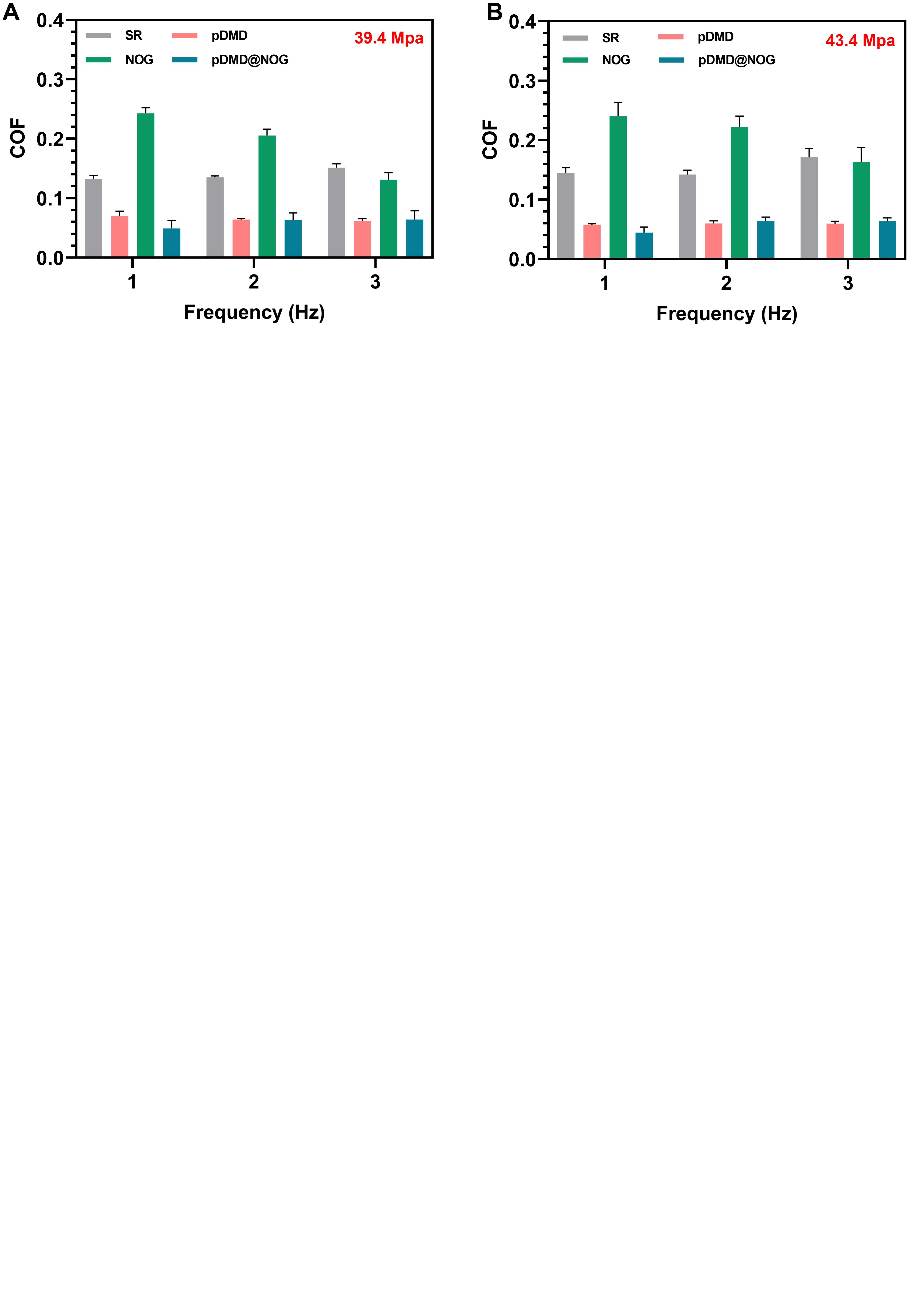
**

Fig. S4. The coefficient of friction (COF) versus frequency plots for PS microspheres in contact with different samples under the pressures of (A) 39.4 and (B) 43.4 MPa.

**
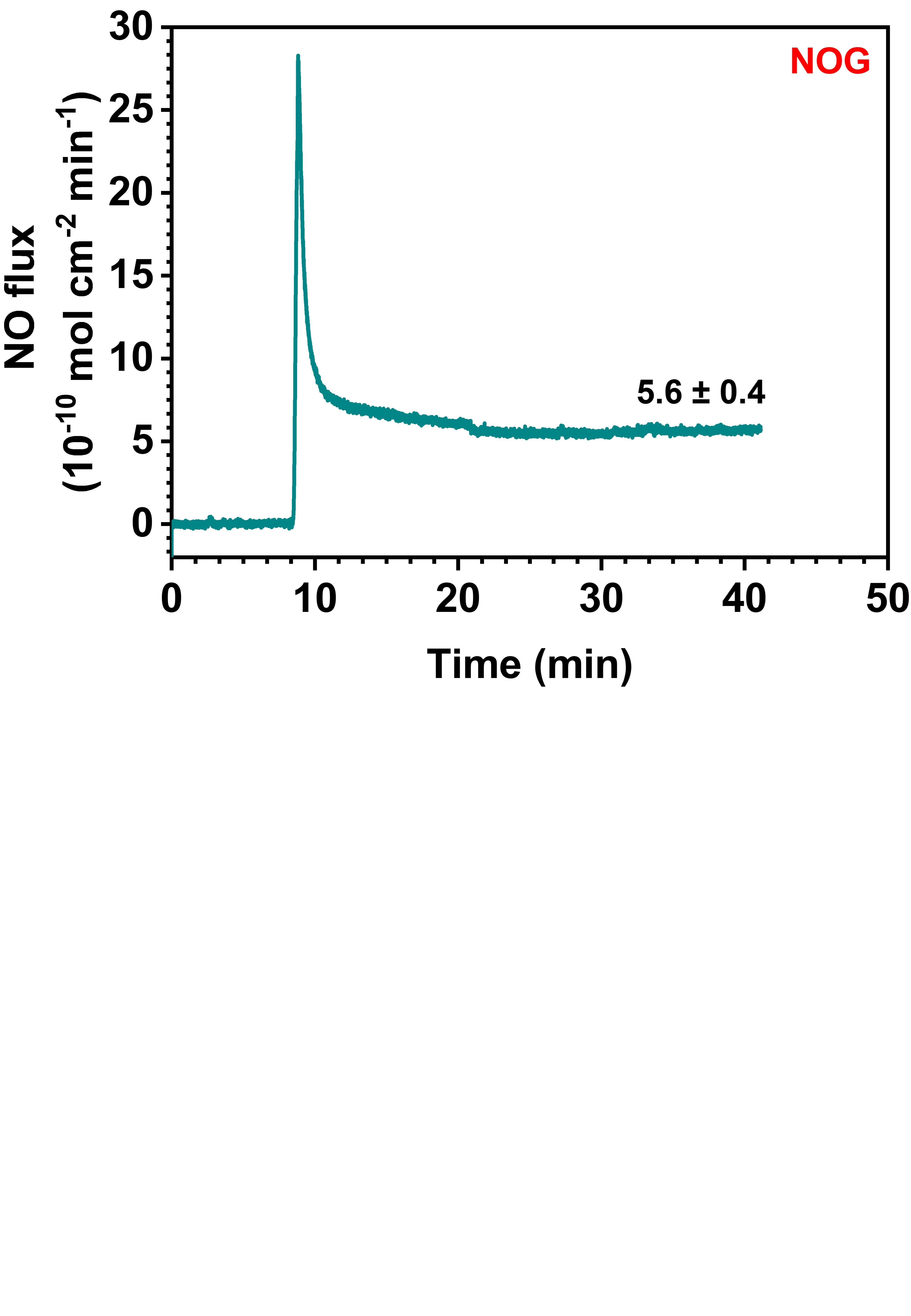
**

Fig. S5. Real-time NO flux from NOG supplemented with NO donor (10 µM of GSNO and 10 µM of GSH).

**
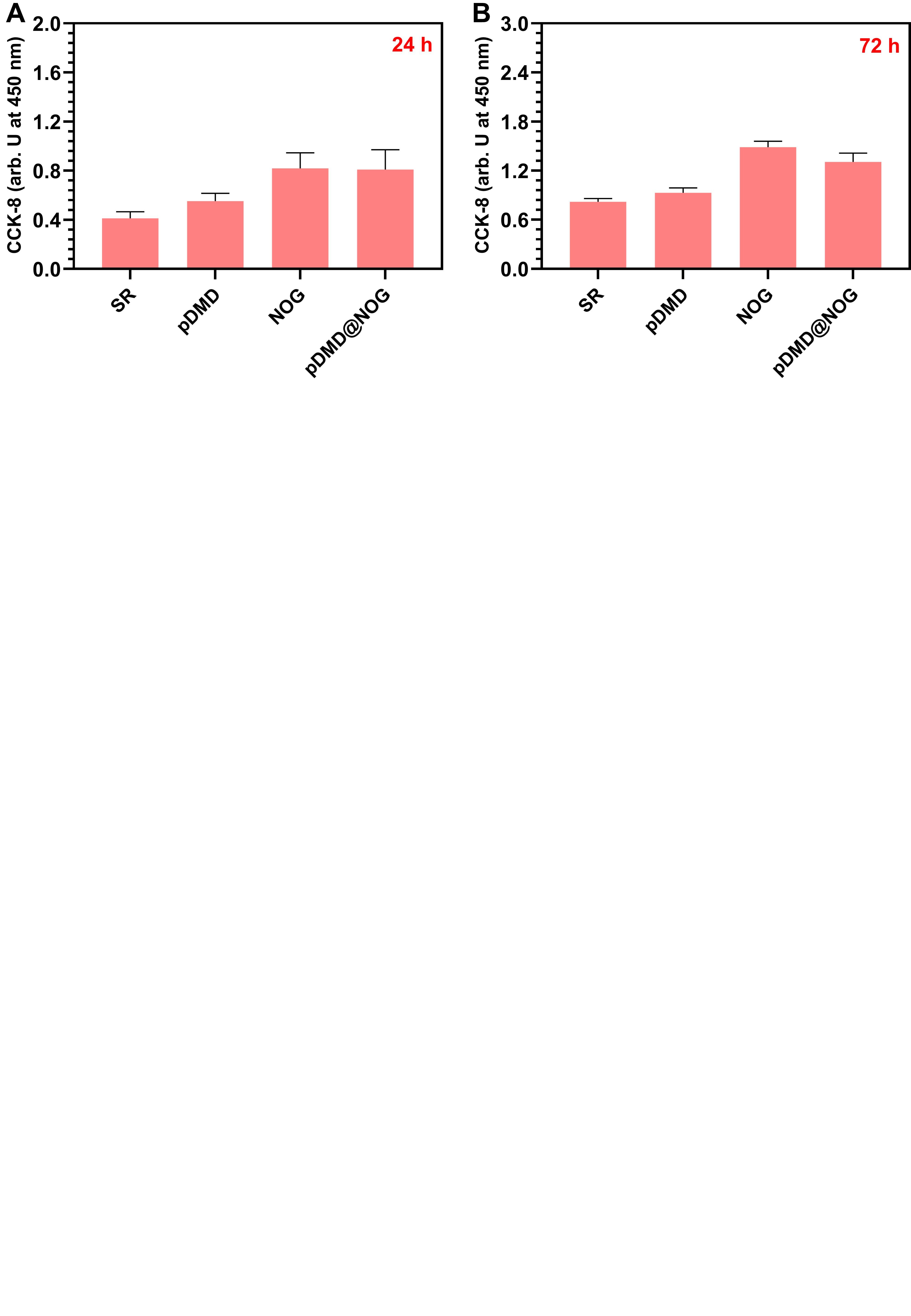
**

Fig. S6. Cell viability after 24 (A) and 72 h (B) of incubation determined using CCK-8 assay.

**
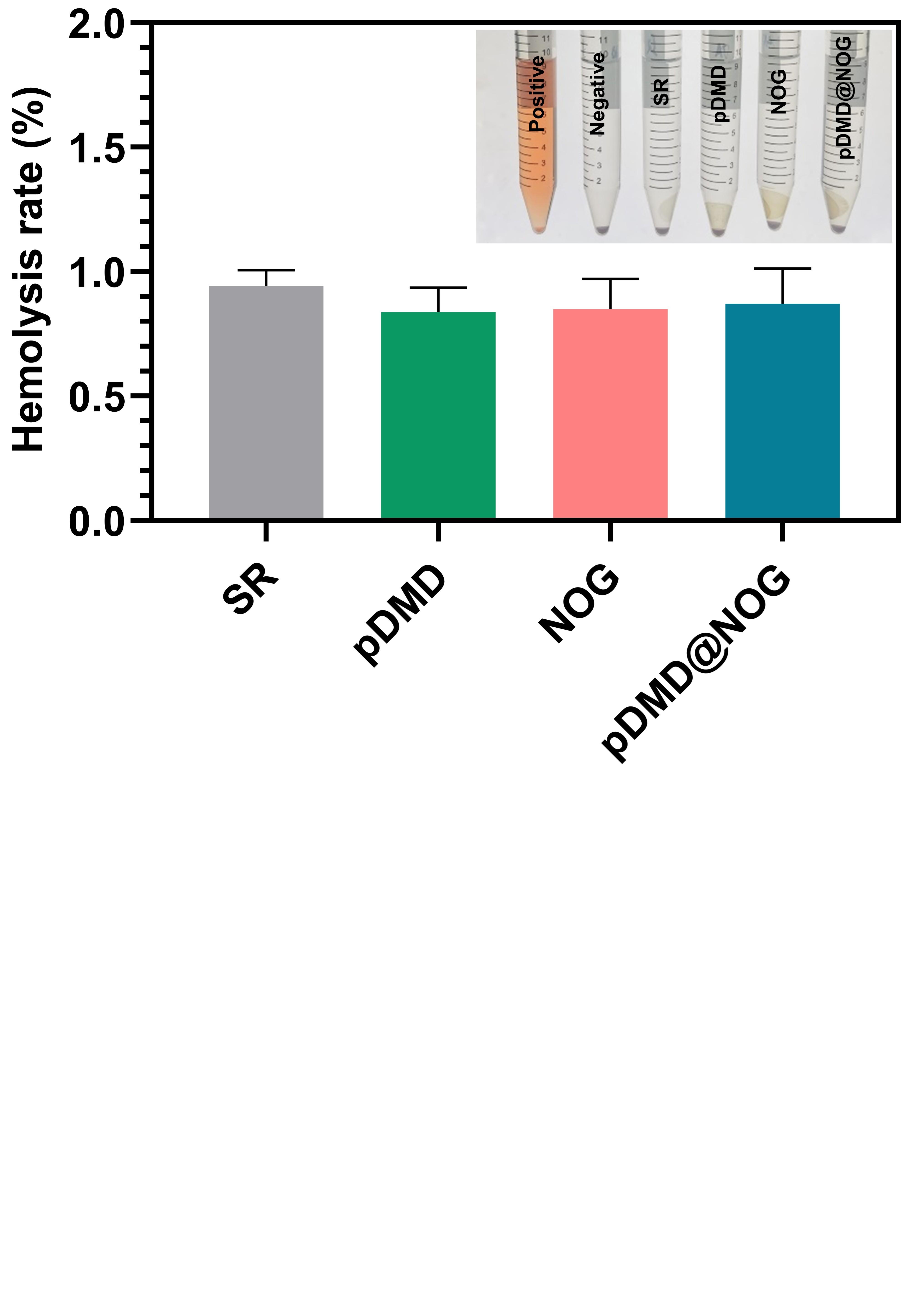
**

Fig. S7. Hemolysis rates evaluation of different samples.


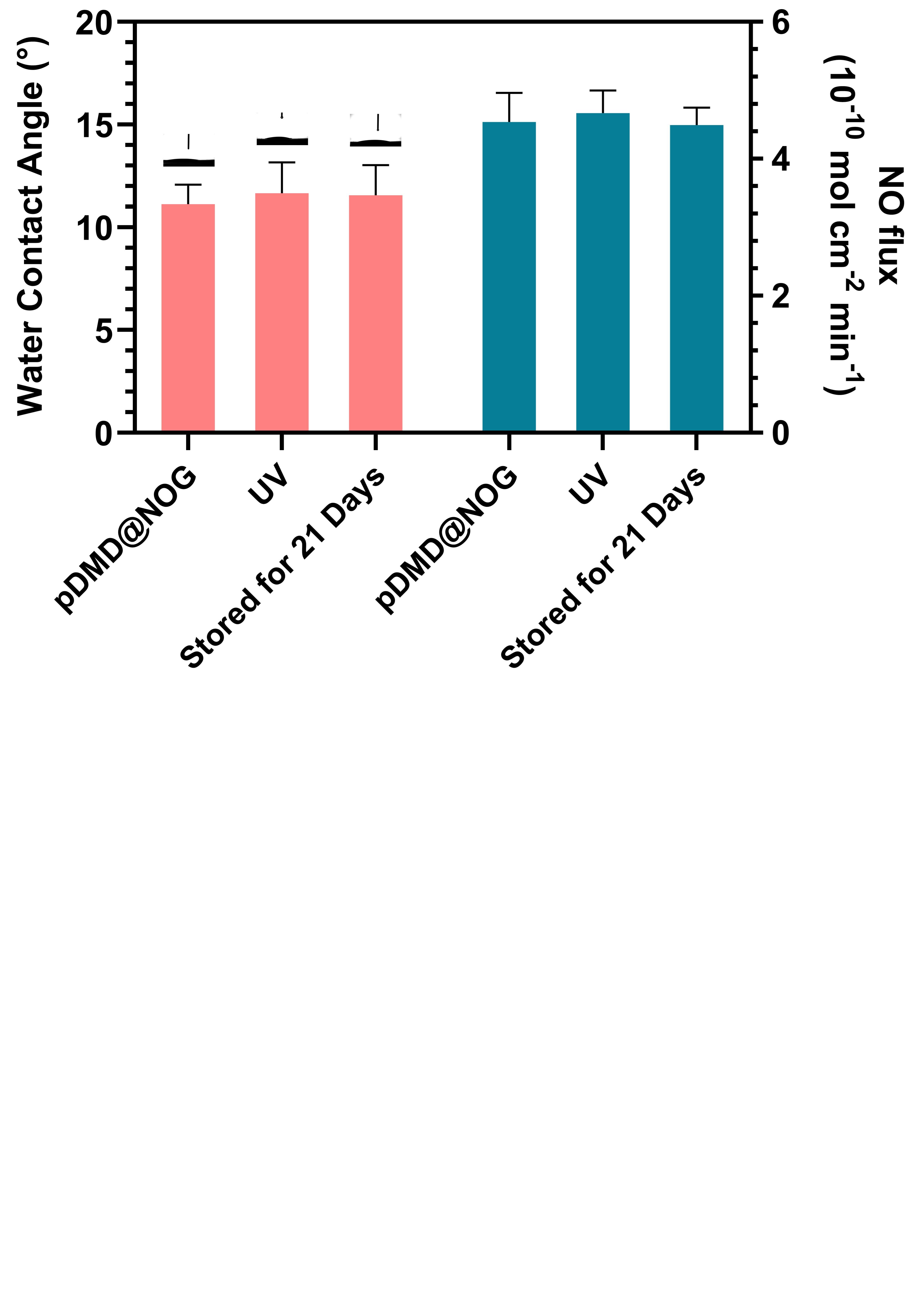


Fig. S8. Water contact angle and NO release rate of pDMD@NOG before and after UV sterilization (284 nm, 300 W, 30 min) and after 21 days of storage.
