# Supplementary figures and images for: Bioinspired Zwitterionic Block Polymer-Armored Nitric Oxide-Generating Coating Combats Thrombosis and Biofouling
Source: Research (Wash D C). 2024 Aug 1;7:0423. doi: 10.34133/research.0423 (PMC11290871; doi:10.34133/research.0423)

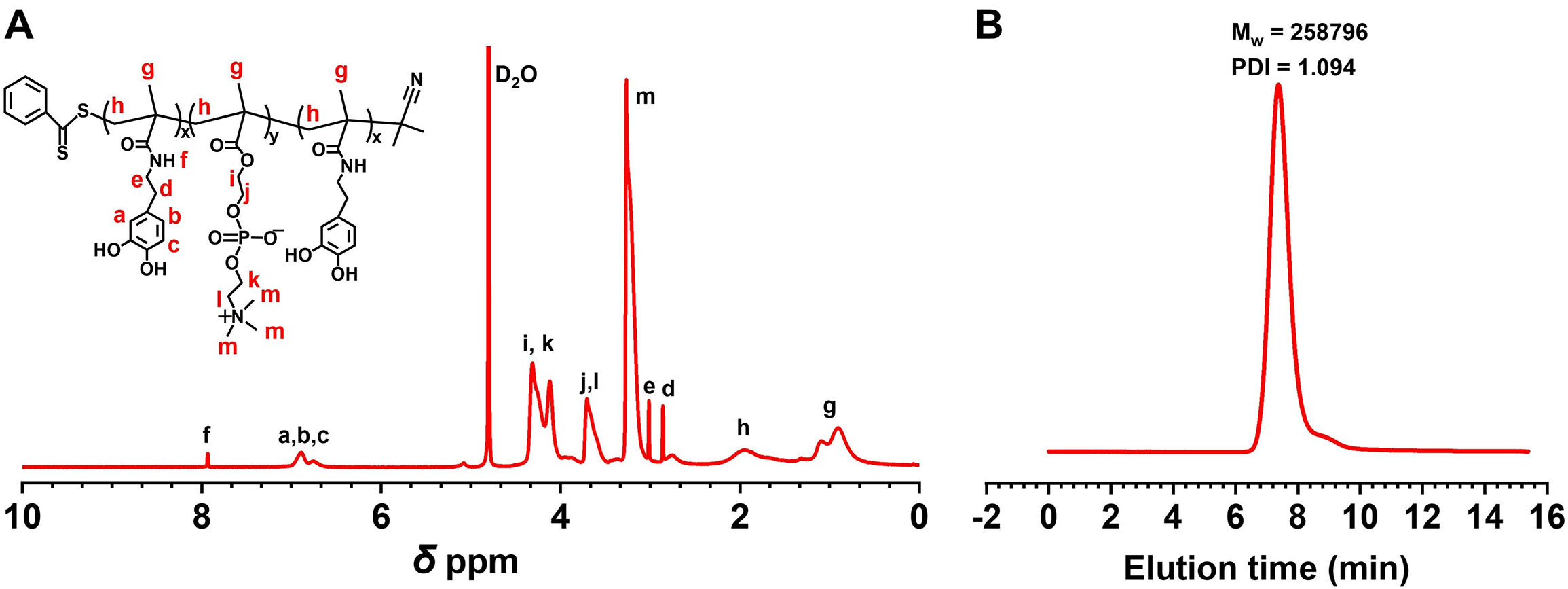

Supplement: Supplementary 1 — Search strategy Figs. S1 to S8 [file research.0423.f1.zip › Fig S1.jpg]

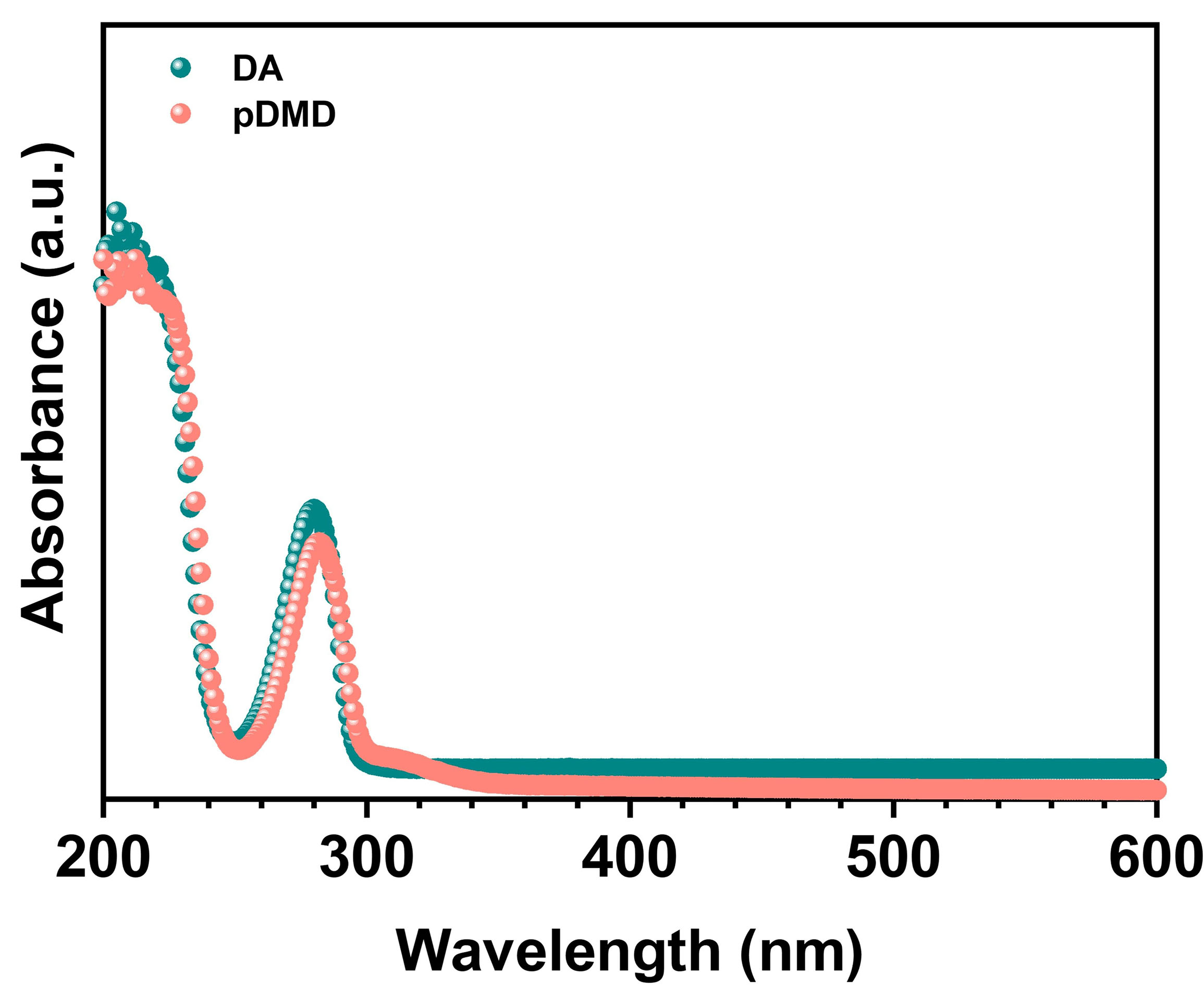

Supplement: Supplementary 1 — Search strategy Figs. S1 to S8 [file research.0423.f1.zip › Fig S2.jpg]

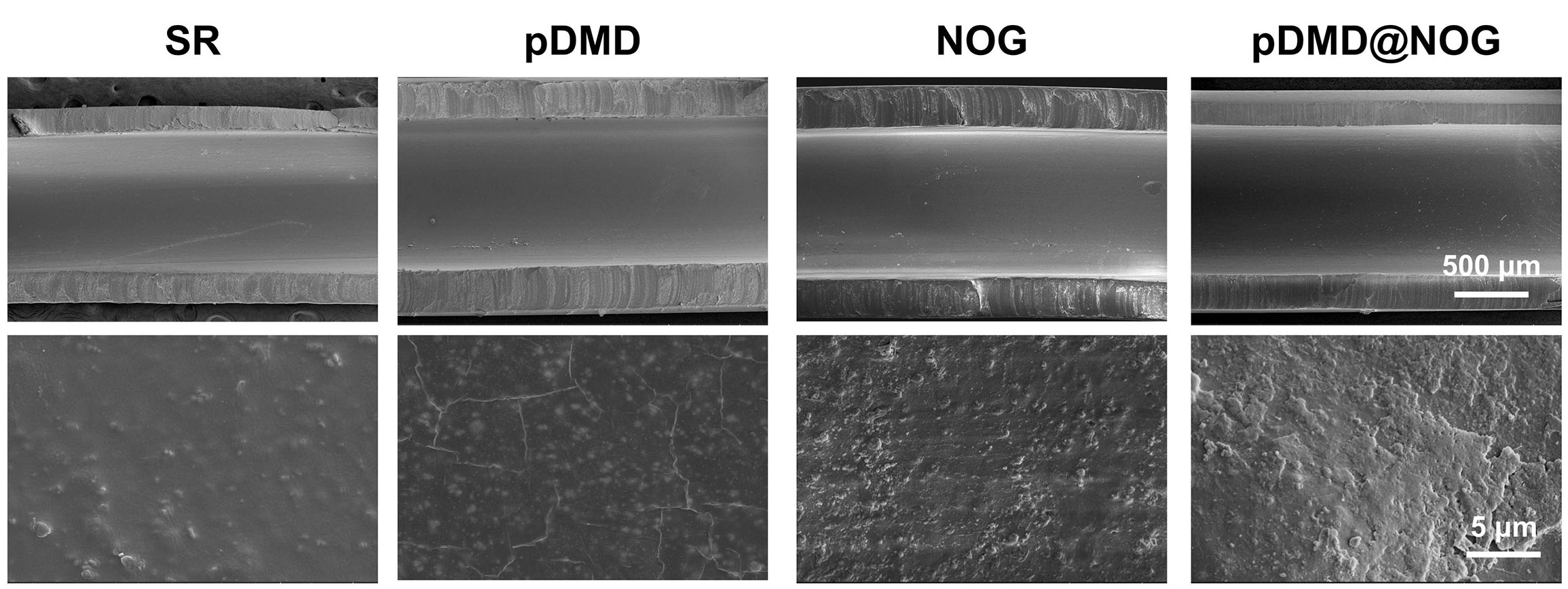

Supplement: Supplementary 1 — Search strategy Figs. S1 to S8 [file research.0423.f1.zip › Fig S3.jpg]

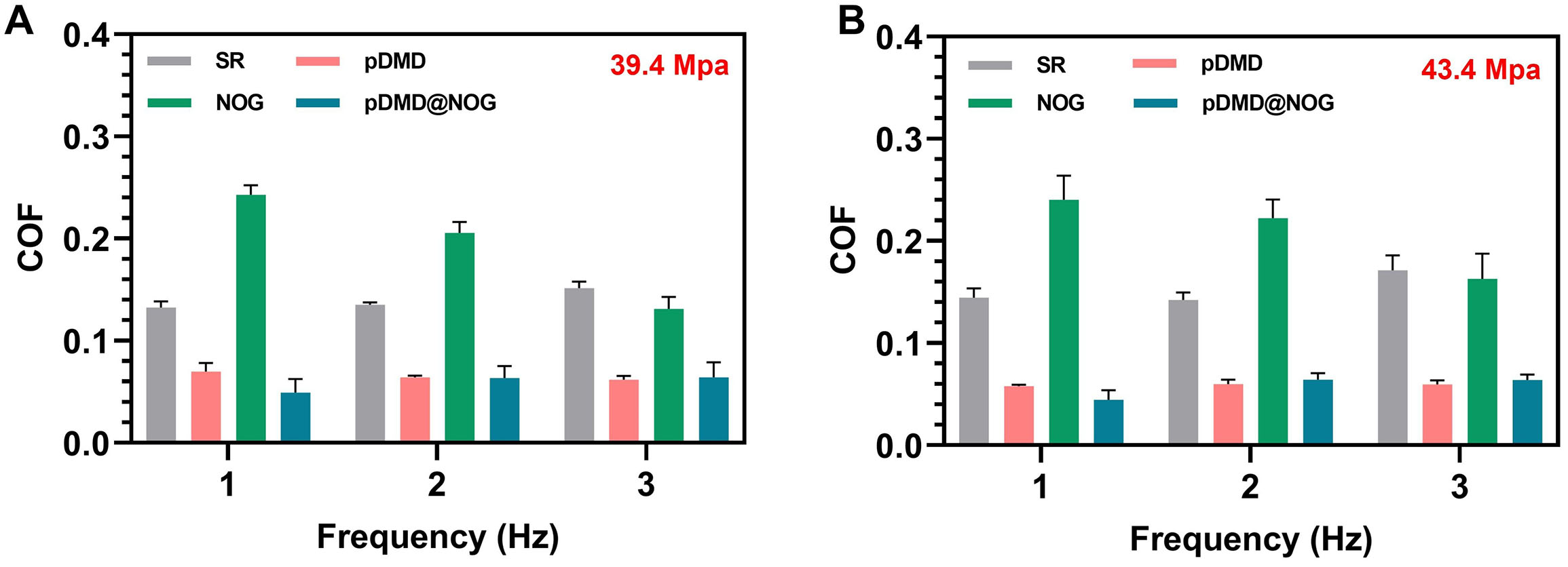

Supplement: Supplementary 1 — Search strategy Figs. S1 to S8 [file research.0423.f1.zip › Fig S4.jpg]

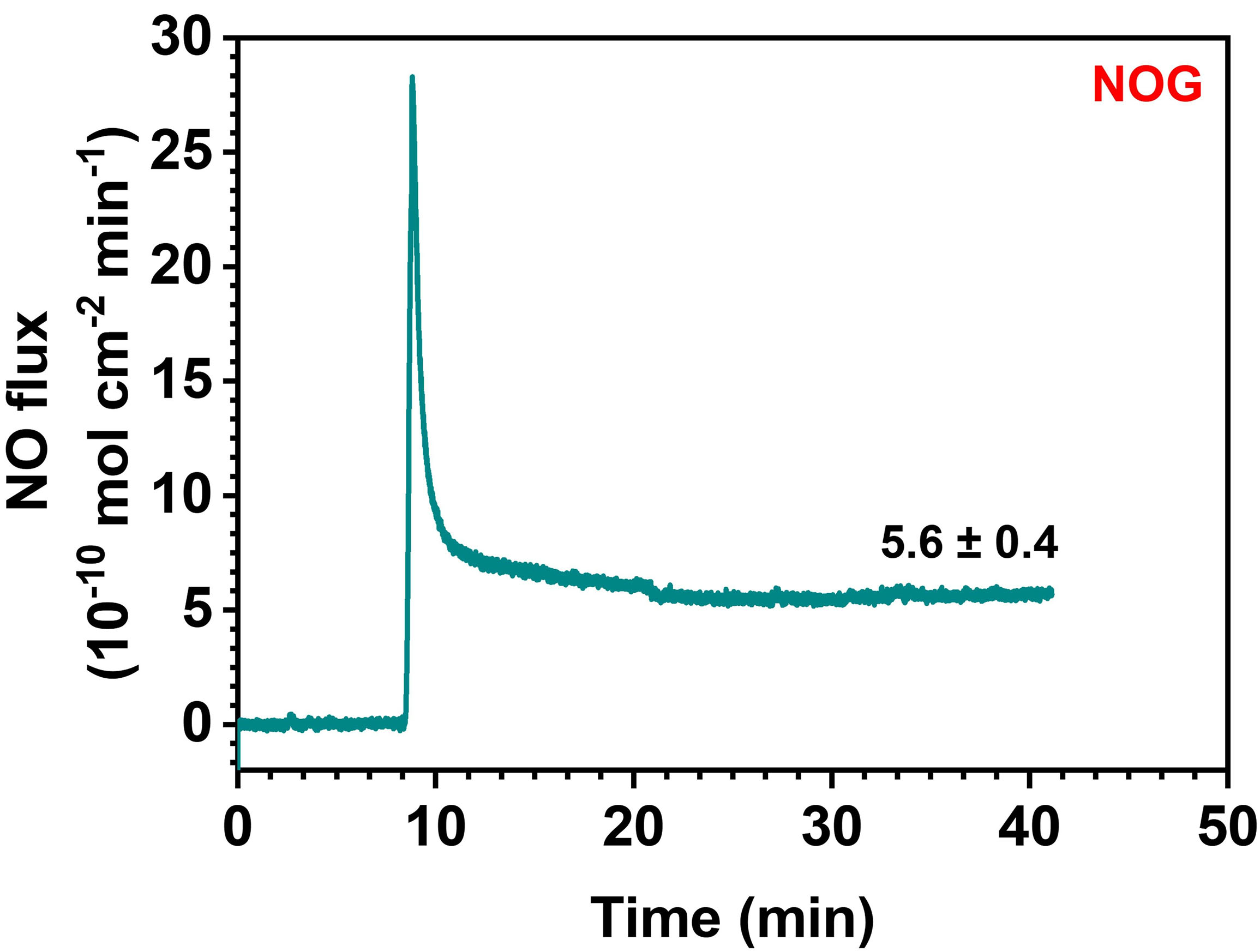

Supplement: Supplementary 1 — Search strategy Figs. S1 to S8 [file research.0423.f1.zip › Fig S5.jpg]

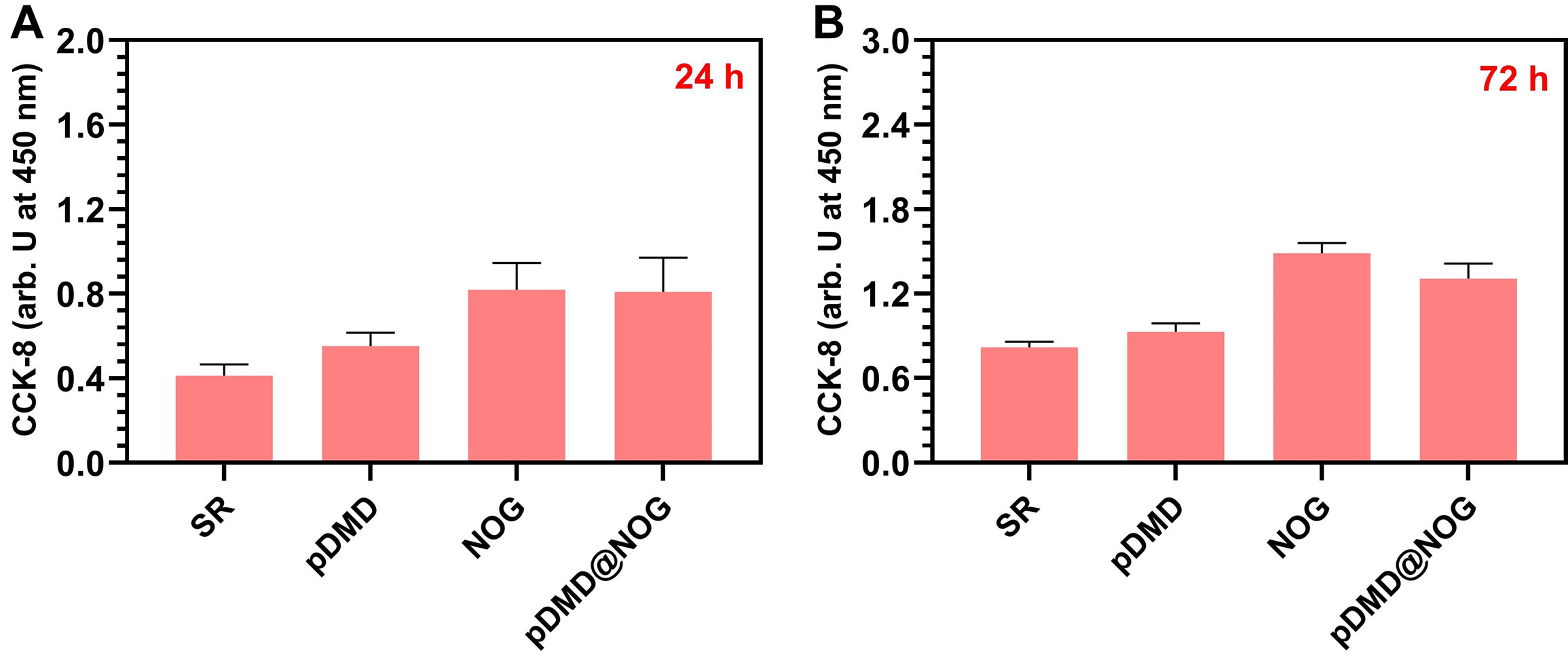

Supplement: Supplementary 1 — Search strategy Figs. S1 to S8 [file research.0423.f1.zip › Fig S6.jpg]

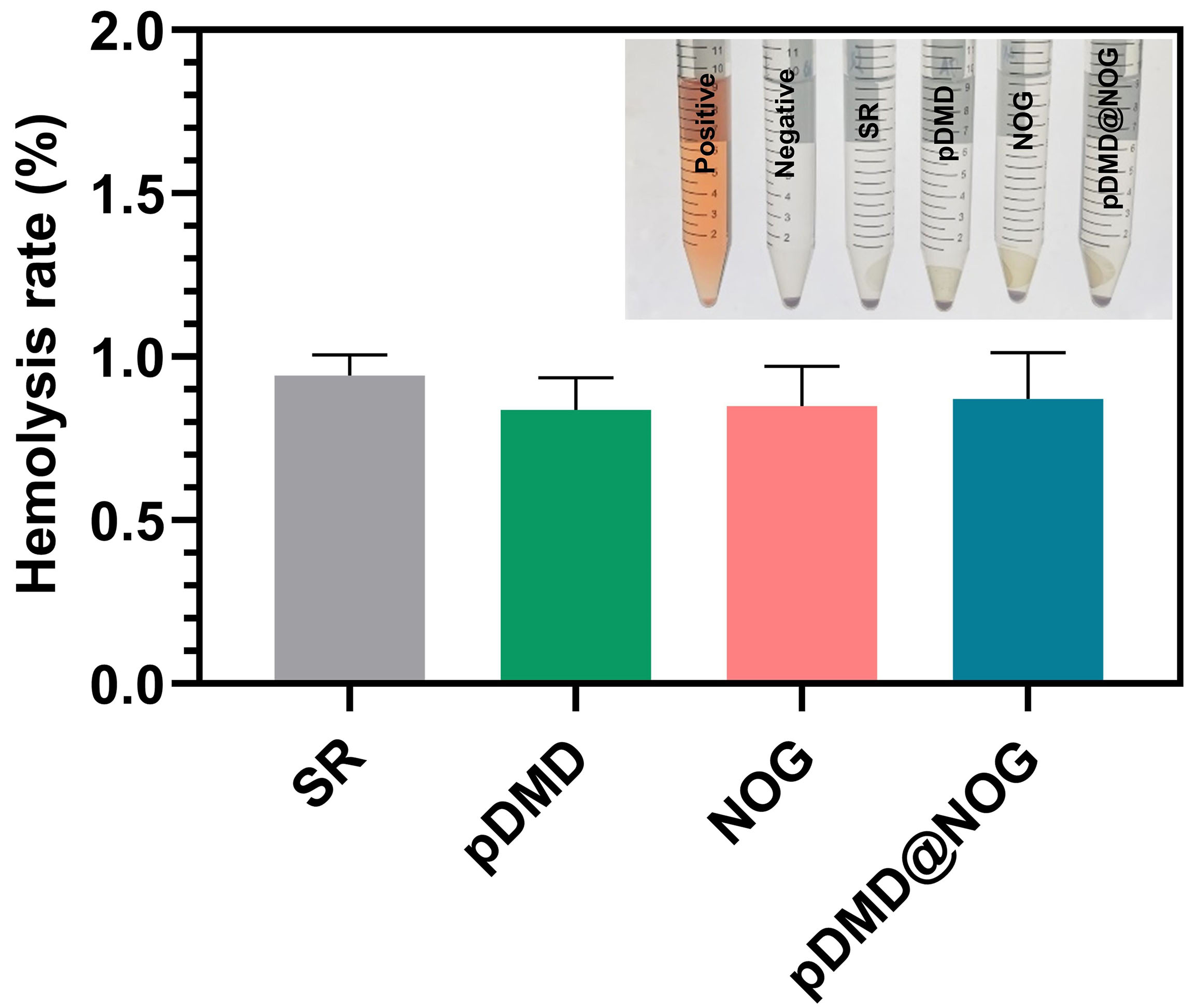

Supplement: Supplementary 1 — Search strategy Figs. S1 to S8 [file research.0423.f1.zip › Fig S7.jpg]

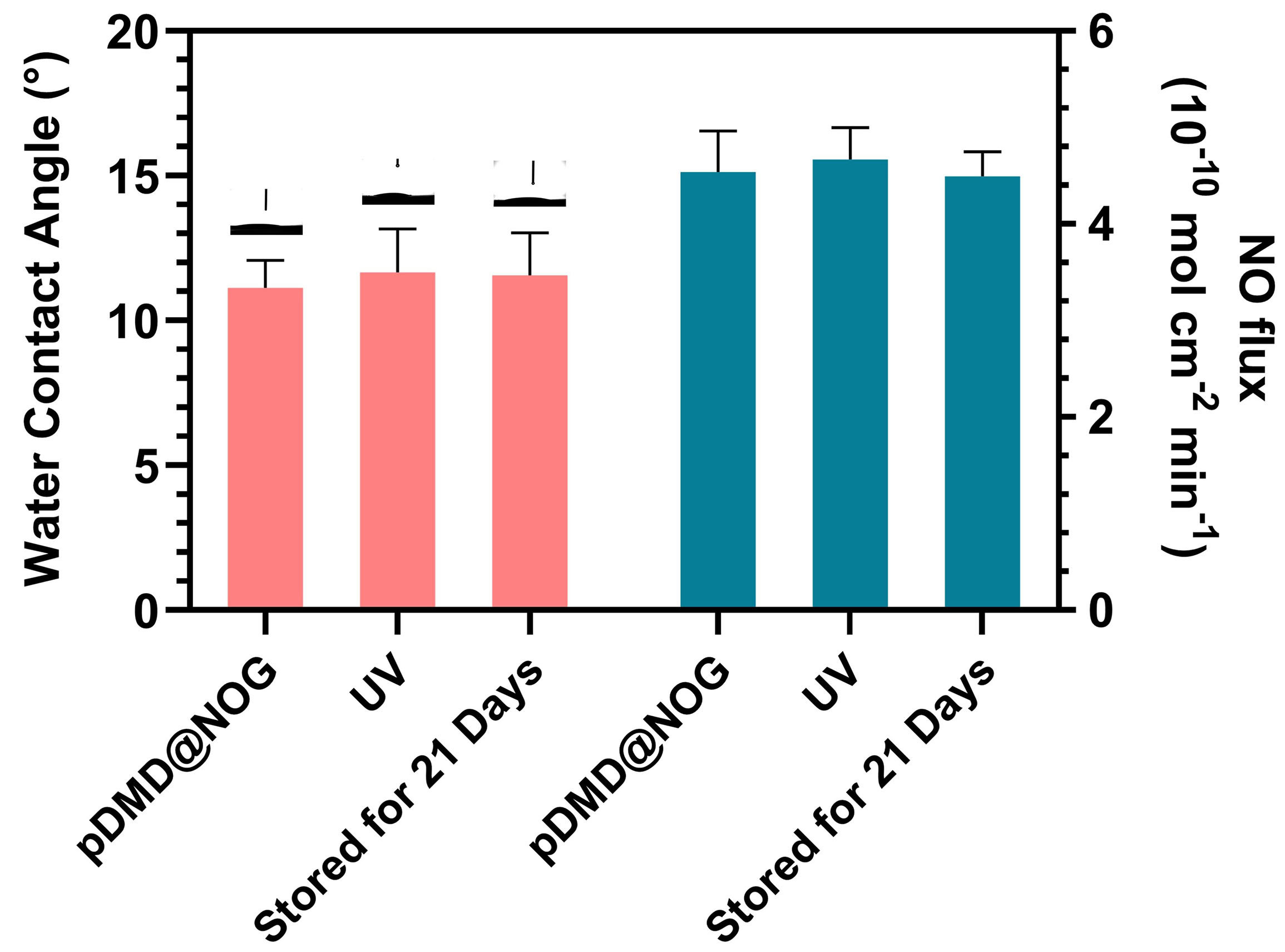

Supplement: Supplementary 1 — Search strategy Figs. S1 to S8 [file research.0423.f1.zip › Fig S8.jpg]
